# Development of High‐Throughput Genomic Resources to Inform White‐Tailed Deer Population and Disease Management

**DOI:** 10.1111/1755-0998.70085

**Published:** 2025-11-26

**Authors:** David Navarro, Emily K. Latch, Anaïs K. Tallon, Caitlin N. Ott‐Conn, Randy W. DeYoung, Daniel P. Walsh, Peter T. Euclide, Chandika R. G., Wes A. Larson, Arun S. Seetharam, Andrew J. Severin, James M. Reecy, Zhi‐Liang Hu, Jay R. Cantrell, Michelle Carstensen, Joe N. Caudell, Charlie H. Killmaster, Mitch L. Lockwood, William T. McKinley, Andrew S. Norton, Krysten L. Schuler, Daniel J. Storm, Jason A. Sumners, W. David Walter, Julie A. Blanchong

**Affiliations:** ^1^ Department of Natural Resource Ecology and Management Iowa State University Ames Iowa USA; ^2^ Behavioral and Molecular Ecology Research Group, Department of Biological Sciences University of Wisconsin‐Milwaukee Milwaukee Wisconsin USA; ^3^ Michigan Department of Natural Resources Wildlife Division Marquette Michigan USA; ^4^ Caesar Kleberg Wildlife Research Institute, MSC 218 Texas A&M University‐Kingsville Kingsville Texas USA; ^5^ U.S. Geological Survey Montana Cooperative Wildlife Research Unit, University of Montana, Wildlife Biology Program Missoula Montana USA; ^6^ Department of Forestry and Natural Resources, Illinois‐Indiana Sea Grant Purdue University West Lafayette Indiana USA; ^7^ Alaska Fisheries Science Center, NOAA Alaska Fisheries Science Center Auke Bay Laboratories Juneau Alaska USA; ^8^ Office of Biotechnology, Genome Informatics Facility Iowa State University Ames Iowa USA; ^9^ Department of Animal Science Iowa State University Ames Iowa USA; ^10^ South Carolina Department of Natural Resources Columbia South Carolina USA; ^11^ Minnesota Department of Natural Resources Wildlife Health Program, 5463 West Broadway Forest Lake Minnesota USA; ^12^ Indiana Department of Natural Resources Bloomington Indiana USA; ^13^ Georgia Department of Natural Resources Wildlife Resources Division Social Circle Georgia USA; ^14^ Texas Parks and Wildlife Department Kerrville Texas USA; ^15^ Mississippi Department of Wildlife, Fisheries, and Parks Jackson Mississippi USA; ^16^ South Dakota Game, Fish and Parks Rapid City South Dakota USA; ^17^ Department of Public and Ecosystem Health Cornell University College of Veterinary Medicine Ithaca New York USA; ^18^ Wisconsin Department of Natural Resources Madison Wisconsin USA; ^19^ Missouri Department of Conservation Jefferson City Missouri USA; ^20^ U.S. Geological Survey, Pennsylvania Cooperative Fish and Wildlife Research Unit The Pennsylvania State University University Park Pennsylvania USA

**Keywords:** DNA microarray, genetic diversity, *Odocoileus virginianus*, population structure, single nucleotide polymorphisms

## Abstract

White‐tailed deer ( *Odocoileus virginianus* ) are the most abundant and widespread cervid in North America. Genetic data are used as a tool to monitor populations and make management decisions for this game species. However, the development and use of genomic tools that can generate a set of markers suitable for longitudinal genomic data collection, whether for management purposes or to study the demographic and evolutionary processes of widely distributed species, have been challenging. This is mainly due to the cost required to fully implement and interpret the data produced. Here, we generated whole genome resequencing data for 44 free‐ranging deer from three regions in their central and eastern North American range and identified over 89 million single nucleotide polymorphisms (SNPs). We used a subset of these SNPs to develop two nested SNP tools, a high‐density array (702,183 SNPs) and a medium‐density array (72,723 SNPs) to support deer and chronic wasting disease (CWD) management and research. SNPs were selected to ensure an even distribution across scaffolds of the reference genome and include SNPs associated with CWD susceptibility. Using genotyping results for 469 deer from 15 states in the US and Mexico generated by the high‐density array and 1335 deer from 18 states generated by the medium‐density array, we assessed genotyping success across different populations and explored some insights into population structure. These genomic tools offer a standard set of markers that will enable researchers and managers to address important questions related to white‐tailed deer and CWD management. Our SNP arrays also offer the opportunity to examine aspects of white‐tailed deer ecology and evolutionary history that were previously difficult to address.

## 1 Background

White‐tailed deer ( *Odocoileus virginianus* ) are the most abundant and widespread species of large mammal in North America (Heffelfinger 2011). Free‐ranging white‐tailed deer are highly adaptable and thrive in diverse landscapes including forests, agricultural areas, and urban environments (Smith 1991). Given large current effective population sizes, high dispersal potential, remarkable capacities for recruitment, and adaptability to new environmental conditions, white‐tailed deer are a good candidate for landscape genetics research to better understand adaptation and how natural populations respond to changing environmental conditions (Long et al. 2005; Lutz et al. 2016; Walter et al. 2009; Webb et al. 2010). However, these characteristics have also made it challenging to develop broadly applicable genetic resources that are informative both at a fine scale and range‐wide, but also emphasise their potential to advance research, conservation and management.

White‐tailed deer shape the ecosystems in which they live by changing habitat composition and structure through browsing, serving as an important food source for predators and scavengers, and facilitating nutrient cycling with cascading effects throughout the community (Waller and Alverson 1997). As one of the most economically and culturally valued resources in North America, white‐tailed deer represent a considerable source of revenue for many state natural resource agencies. An estimated 11.5 million individuals spent 135 million days pursuing big game like white‐tailed deer in 2022, contributing to a $400 billion annual wildlife‐related recreation economy in the US (USDOI and USFWS 2022). In parallel, white‐tailed deer can cause negative economic and ecological consequences associated with habitat damage, vehicle collisions, and transmission of several diseases (Côté et al. 2004). Threats to wildlife populations often act synergistically, and in combination with genetic factors (Ceballos et al. 2017), make it essential to monitor genetic diversity and population structure to inform conservation and management decisions. For example, links between gene flow and disease prevalence, and susceptibility have been shown in white‐tailed deer (Blanchong et al. 2008; Lang and Blanchong 2011; Kelly et al. 2014; Chafin et al. 2020). Therefore, consistent genetic data may help identify high‐risk populations and monitor changing deer dispersal.

White‐tailed deer are hosts for chronic wasting disease (CWD), a highly infectious and fatal transmissible spongiform encephalopathy that has been rapidly emerging in cervid populations and is, therefore, of particular concern to wildlife researchers and managers. Originally discovered in 1967, this progressive neurodegenerative disease is caused by misfolded prions with high β‐sheet content that propagate via templated conformational conversion of the cervid's normal prion protein isoform (Safar et al. 1993; Pan et al. 1993; Kocisko et al. 1994). Since its discovery, CWD has increased in prevalence and distribution, with infected free‐ranging and captive deer reported in 35 US states and 4 Canadian provinces, as well as in Norway, Finland, Sweden and South Korea (Escobar et al. 2020; USGS 2024; CDC 2023). With no cure for CWD, current options for mitigating the spread of CWD are limited. Several free‐ranging populations of cervids have been shown to be impacted by CWD (Monello et al. 2014; Edmunds et al. 2016; DeVivo et al. 2017), and there is growing concern of potential risks to human health (Osterholm et al. 2019).

Managing white‐tailed deer and associated infectious pathogens is challenging, given the central role of white‐tailed deer in ecosystems across North America and their importance as a natural resource. Ecological and natural history research has been critical for understanding deer biology and their relationship to local ecosystems. Genetic assessments have complemented field studies to address important questions about white‐tailed deer ecology and have provided valuable insights for management. For instance, genetic studies have identified signatures of historical translocation efforts (Leberg and Ellsworth 1999; DeYoung et al. 2003), revealed impacts of isolation or confinement on genetic diversity (Blanchong et al. 2013; Latch et al. 2021), and documented relationships between population genetic structure and landscape features that shape deer movement patterns and disease spread (Blanchong et al. 2008; Kelly et al. 2010). Furthermore, genetic methods have contributed to a better understanding of deer reproductive ecology, social structure, and behaviours relevant for disease and population management (Blanchong et al. 2007; DeYoung et al. 2009; Grear et al. 2010). Genetic associations between disease susceptibility and subsequent selective impacts on populations have also been explored. The most notable efforts are related to CWD and the prion gene, *PRNP*, which has been associated with CWD progression in white‐tailed deer (Johnson et al. 2006; Robinson, Samuel, O'Rourke, and Johnson 2012; Robinson, Samuel, Lopez, and Shelton 2012; Blanchong et al. 2009).

Except for targeted sequencing of the PRNP gene, most of the studies described above are constrained by the limitations of microsatellite markers. Microsatellite markers have been the conventional method of choice for population genetic studies of white‐tailed deer. Microsatellite markers contain many alleles per locus and can provide up to twelve times more information than a single nucleotide polymorphism (SNP) locus (Liu et al. 2005). However, microsatellite genotyping panels can be difficult to develop, replicate amongst studies, and standardise amongst laboratories (Hoffman and Amos 2005), and most panels are constructed to investigate neutral genetic variation outside of coding regions (Selkoe and Toonen 2006). The small amount of information provided by an individual SNP can be overcome by advances in next‐generation sequencing technologies and associated reductions in sequencing costs, which facilitate genotyping thousands or hundreds of thousands of SNPs across the genome at a comparable cost to microsatellites (Puckett 2017). Sequencing more loci spread across the genome has increased precision in population parameter estimates generated using SNP data, when compared to microsatellite markers (Flanagan and Jones 2019; Zimmerman et al. 2020; Laoun et al. 2020; Galla et al. 2022). Additionally, SNPs are effective for addressing common applied genetic challenges, such as genotyping degraded DNA (Von Thaden et al. 2017), quantifying species hybridization (Stronen et al. 2022), and monitoring invasive species (Sjodin et al. 2020). Finally, because SNPs are most often genotyped, using sequencing data or oligonucleotide sequences and established bioinformatic pipelines and are genotyped based on specific nucleotide calls rather than allele sizes, genotype data are more easily replicated and comparable across laboratories. This can facilitate collaborative investigation of wide‐ranging species by enabling data integration across larger spatial and temporal scales (Seeb et al. 2011).

Combining sets of pre‐identified and informative SNPs into easily accessible panels provides opportunities to quantify population structure, measure local adaptation, and identify and track disease‐associated genetic variants in captive and wild white‐tailed deer populations. To create an ideal SNP panel for white‐tailed deer, molecular markers must be distributed throughout the genome, including in areas of high variability and genes of interest. Previous studies focused on wildlife have often applied SNP panels created for livestock or other model organisms. However, these arrays may be affected by ascertainment bias, often resulting in a fraction of the SNPs successfully genotyping (Haynes and Latch 2012; Miller et al. 2012; Kharzinova et al. 2015). Other studies have begun to develop genomic resources, such as SNP sets (Chafin et al. 2021; Fuller et al. 2020), chromosome‐level assemblies (London et al. 2022; Poisson et al. 2023), and restriction site associated DNA (RAD)‐sequencing (Gervais et al. 2019) for white‐tailed deer and other cervids. Yet, these tools may not provide data that are directly comparable or even fully repeatable due to project‐specific development and reliance on reduced representation approaches for marker discovery. A standardised set of markers that considers the high abundance and broad distribution of white‐tailed deer would enable data sharing across projects and facilitate in‐depth studies of selection and phylogeographic history.

The objective of this study was to develop genomic tools to support deer and CWD management. To accomplish this, we generated whole‐genome resequencing data for white‐tailed deer from a broad geographic area across their eastern and central range. Using high‐quality SNPs from the resequencing data, we developed two genomic tools to survey variation across the white‐tailed deer genome, a high‐density array (OVSNP600) with ~700,000 SNPs and a medium‐density array (OVSNP60) with ~70,000 SNPs. We genotyped these SNPs in over 1900 deer to characterise genetic diversity both genome‐wide and at CWD‐associated loci and assessed patterns of genetic structure. By partnering with federal and state agencies, we were able to obtain a large number of samples from across a substantial portion of the species range to develop genomic tools that comprehensively survey the genome‐wide variation across the range of white‐tailed deer. The results of this study are an important first step toward the development of a standardised set of SNP markers for coordinated conservation and management of free‐ranging white‐tailed deer.

## 2 Methods

### 2.1 Deer Resequencing

Samples from 45 free‐ranging, male white‐tailed deer originating from 3 geographic areas in the United States representing the ranges of 3 named subspecies (Heffelfinger 2011), including 17 hunter‐harvested deer from southeast Iowa (*O. v. macrourus*), 16 deer from south Texas (*O. v. texanus*), and 12 deer from the Pisgah Game Preserve in North Carolina (*O. v. virginianus*), were used for whole genome sequencing in this study. We extracted DNA using the Qiagen DNEasy kit (QIAGEN, Valencia, CA) according to the manufacturer's protocol, quantified DNA concentrations using a Qubit fluorometer, and assessed DNA quality via agarose gel electrophoresis. We submitted ≥ 2 μg of genomic DNA from each individual to the Iowa State University DNA Facility, which constructed PCR‐free libraries. Samples were indexed, pooled, and run together on 24 lanes for 2 × 100 paired‐end sequencing on an Illumina HiSeq 3000.

### 2.2 Variant Calling of Resequencing Data

Sequencing data were obtained as fastq files and checked for quality using the program FASTQC (version 0.11.5). Upon finding the quality scores to be satisfactory, and without adapter contamination, we processed reads using GATK (version 4.0) (DePristo et al. 2011; Van Der Auwera et al. 2013). Raw reads were converted to sequence alignment map (SAM) files using the FastqToSam function in Picard Tools. Illumina adapters were screened and marked using the function MarkIlluminaAdapters. Reads were aligned to the white‐tailed deer reference genome (GCA_002102435.1) using the BWA mem algorithm (v 0.7.17) (Li and Durbin 2009). Mapped and unmapped reads were merged to the genome using the function MergeBamAlignment utility. SNPs were only retained if they had a minimum average read depth of 10 and a non‐reference allele count of at least 10, ensuring that low‐frequency or potentially spurious variants due to mapping bias were removed. Finally, read groups and sample names were added using the function AddOrReplaceReadGroups utility. We performed a first round of SNP calling using GATK HaplotypeCaller (v 4.0.4.0) (McKenna et al. 2010) using default parameters, processing each scaffold independently to speed up this step. We excluded one sample from the final file, which had the lowest coverage on average and was missing over 36% of the SNP data, resulting in a total of 44 samples retained. We subjected the first round SNPs to stringent hard filtering based on the following criteria: SNPs with quality by depth score < 2.0, Phred‐scaled *p*‐value using Fisher's exact test to detect strand bias >60.0, mapping quality <45.0, root mean square of the mapping quality < −12.5, Mann–Whitney rank sum test for mapping qualities < −8, and depth > 920.

To improve the confidence in the SNP selection, we performed another round of filtering on the hard‐filtered SNPs using VCFtools (v 0.1.14) (Danecek et al. 2011). As a result, only SNPs with the following criteria were retained: 0% missing data, minor allele counts ≥ 3, sites with quality value > 30 and minimum depth (DP) of 3. We then used this filtered SNP dataset to generate a table of recalibrated bases using the BaseRecalibrator tool from GATK and applied it to the initial binary alignment map (BAM) files using ApplyBQSR. We performed a second round of SNP calling using the recalibrated BAM files following the same steps as described in the first round. We subjected the final variant call format (VCF) file to the same stringent hard‐filtering using GATK followed by VCFtools filtering criteria as before. Due to the large number of SNPs in the filtered final file, we prepared an additional low‐density SNP file using the VCFtools thin function (randomly selecting 1 SNP every 100 bp). We generated summary statistics for both VCF files with the stats function in BCFtools (v 1.9‐womp5fh)(Danecek et al. 2021). We phased the low‐density SNP file using Beagle (v 28Sep18.793) with default parameters and used VCFtools to calculate depth of coverage, missing site frequency for each individual, and the number of transitions and transversions.

### 2.3 Selection of SNPs for the OVSNP600 From Resequencing Data

The OVSNP600 was developed using the Axiom myDesign genotyping Array Kit (Thermo Fisher Scientific, ThermoFisher) in a 96‐array layout to include ~600,000 SNPs obtained from the resequencing data (57,950,917 SNPs). The first step in selecting SNPs was to identify approximately 5 million SNP probes (i.e., SNPs and associated 35 nucleotide upstream and downstream sequences) that ThermoFisher would evaluate for the probability of success on the array. SNPs were further thinned based on the following criteria: minor allele frequency ≥ 0.05, mean depth across individuals of at least 10, and present in at least 10 individuals. Further, all transversions were removed to maximise the number of SNPs that could fit on the array (as transversions require more space than transitions) and all scaffolds were represented. Following this filtering, 5,242,424 SNP probes (along with 3172 37‐nucleotide non‐polymorphic control sequences) were sent to ThermoFisher where SNP probes were assigned pconvert scores. The pconvert score is a measure of the relative probability of a given probe's success relative to the other probes under consideration, based on both probe thermodynamics and the number of probe hits found in the reference genome. Pconvert scores were placed into categories of “not recommended” (0 < pconvert < 0.4), “neutral” (0.4 ≤ pconvert < 0.6) and “recommended” (0.6 ≤ pconvert ≤ 1.0). Ultimately, the final probesets for the OVSNP600 were selected to ensure an even distribution across the 6703 scaffolds of the white‐tailed deer genome (GCA_002102435.1) and a high likelihood to be successfully genotyped (pconvert score ≥ 0.72). One marker at codon 96 in the *PRNP* gene was tiled with 14 probesets. This process yielded 702,182 markers for the OVSNP600 array.

### 2.4 Selection and Screening of Samples for the OVSNP600

To screen the OVSNP600, we collected 480 samples from 15 U.S. states and 2 Mexican states across the white‐tailed deer's eastern, central, and southern North American range (Table S1). High‐quality tissue samples were taken from roadkill, hunter‐harvested deer, or live‐captured deer. Tissue samples were either directly frozen or stored in 95% ethanol or lysis buffer until DNA extraction. Sampling efforts also included DNA extracts from archives within our target areas. Metadata was obtained, when possible, for each sample and included age, sex, and location. Following guidelines from Thermo Fisher Scientific to create the arrays, we only used samples in which CWD was not detected using validated assays. Genomic DNA was isolated from tissue samples using the QIAGEN DNeasy kit according to the manufacturer's protocol.

Quality screening of DNA extracts for the OVSNP600 followed Thermo Fisher Scientific guidelines in the Microarray Research Services Guide. DNA extracts were quantified using a Qubit 4.0 Fluorometer (Invitrogen, Carlsbad, CA). Samples that were above the minimum concentration of 10 ng/μL were then evaluated for molecular weight quality of the DNA by electrophoresis (1% agarose gel) to assess fragment size and degradation. Using a 100 bp ladder as a reference, quality samples with approximately 90% of the DNA greater than 10 Kb, with minimal streaking, were considered for genotyping. Genotyping was performed by Thermo Fisher Scientific and genotypes were called using the Axiom Analysis Suite software (v 5.1.1 Thermo Fisher Scientific), following best practices. A VCF file with allelic calls for all SNPs was exported from the Axiom Analysis Suite and used for all downstream analyses.

### 2.5 Selection of SNPs for the OVSNP60 From the OVSNP600 Data

The OVSNP60 was developed using ThermoFisher's Axiom myDesign genotyping Array in a 384‐array layout. Selection of the SNPs for the OVSNP60 began with the subset of the ‘Best and Recommended’ SNPs included on the OVSNP600 (517,261 SNPs). Probesets were retained based on cluster quality; we kept those that fell in the Poly High Resolution conversion type and that exhibited high cluster quality based on Fisher's Linear Discriminant values (FLD > 4.5), which indicate well‐separated and narrow clusters. These filtering steps retained 473 K probesets. From these, we selected 72,723 probesets with an even distribution across the 6703 white‐tailed deer reference genome scaffolds. We chose not to apply any targeted marker selection criteria related to informativeness for specific downstream analyses (e.g., genetic diversity, *F*_ST_, etc.; Rosenberg et al. 2003; Rosenberg 2005; Armstrong et al. 2025; Solari et al. 2025) to avoid high‐grading biases and retain broad utility of the panel, though these targeted approaches can be useful for specific applications.

### 2.6 Selection and Screening of Samples for the OVSNP60

We collected 1462 samples from 19 U.S. states across the white‐tailed deer's eastern and central range for screening the OVSNP60. Sample criteria were identical to the OVSNP600, although the target DNA concentration needed for the OVSNP60 increased to 17 ng/μL. Genomic DNA was isolated from tissue samples using the QIAGEN DNeasy kit according to the manufacturer's protocol, except for adjustments to two tissue types that were submitted. The first were muscle tissue samples which required double the Proteinase K and incubation time and half the elution volume for sufficient DNA yields. The second tissue type was ear punch samples collected using the Allflex tissue sampling kit (Allflex Merck & Co. Inc., Rahway, NJ), which yielded sufficient DNA concentrations only when using the Allflex sample preservation buffer in the lysis digestion step of the DNeasy protocol.

Screening of DNA extracts for the OVSNP60 also followed ThermoFisher guidelines in the Microarray Research Services Guide and screening steps were identical to the OVSNP600. A total of 1534 samples were included on the OVSNP60: 1462 unique samples from 19 states (samples included by state can be found in Table S1).

We also included 72 samples for various quality control efforts including 61 randomly selected samples that were duplicated on the OVSNP60 arrays, representing all 19 states, and 5 samples that were genotyped on both the OVSNP600 and the OVSNP60. Genotyping and locus filtering were the same as described for the OVSNP600. We compared the genotypes obtained from duplicated samples by tabulating genotype match percentages, and visualising multilocus genotype similarity using a PCA to compare duplicated samples.

### 2.7 CWD Associated SNPs

The *PRNP* gene provides instructions for making the prion protein (PrP). CWD results from a misfold of PrP, which accumulates in the brain and other tissues and is capable of converting other normal PrP into the abnormal form. Variations of the *PRNP* gene have been associated with CWD progression in white‐tailed deer. For example, mutations of the PRNP at gene codons 95 (CCA/CAT; Q → H), 96 (GGT/AGT; G → S), and 116 (GCA/GGA; A → G) were previously found to delay the progression of CWD (Johnson et al. 2003; Heaton et al. 2003; Otero et al. 2019). SNPs located at these variable sites, believed to be associated with CWD progression, were included on both arrays. To increase the chance of genotyping success, 15 probe sets were included to target the *PRNP* SNP at codon 96 in the OVSNP600. Additional *PRNP* SNPs were included on the OVSNP60, with four probesets included to target codon 96, 2 probesets for codon 95, and two probesets for codon 116. In addition, probesets for 55 SNPs located outside the *PRNP* gene, which were previously identified as possibly associated with CWD infection status in captive deer, were also included on the OVSNP60 (Seabury et al. 2020).

The use of these *PRNP* SNPs is complicated by the presence of an unexpressed processed pseudogene (*PRNPy*) (Brayton et al. 2004) with high similarity to the functional *PRNP* gene in some animals. If the presence of the pseudogene influences the genotyping results it could limit the reliability of these SNPs as indicators of *PRNP* variation. ThermoFisher uses the two allele signal intensities, size and contrast, of a SNP to assign confidence scores and creates SNP cluster plots for each probeset. Genotype calls are made using these plots depending on the genotype intensity cluster (AA, AB, or BB) to which each sample most likely belongs (Figure S1). Confidence values that are above the confidence score threshold are assigned as a nocall. The genotype ellipses for the codon 96 SNP refer to high‐confidence genotype calls of AA, AG, and GG. To determine how the pseudogene may be affecting the array genotyping results for this locus we sequenced the functional *PRNP* and pseudogene *PRNPy* in a subset of 96 white‐tailed deer included on the OVSNP60 using Sanger sequencing. We targeted samples whose genotypes at the codon 96 SNP were nested between the AG and AA ellipse (all of which were genotyped as AG, *n* = 22), within or above the AA ellipse (*n* = 11), within or above the AG ellipse (*n* = 43) and within or above the GG ellipse (*n* = 20). Following similar methods to O'Rourke et al. 2004, PCR amplification of the functional *PRNP* gene was performed using the forward primer *PRNP*‐223 (5′‐ACACCCTCTTTATTTTGCAG‐3′) and PCR amplification for the *PRNPy* pseudogene was done using forward primer 379 (5′‐AAGAAAATTCCTGAGAGAGCAT‐3′). These primers exclusively target the functional gene (830 bp) and pseudogene (950–974 bp), respectively. PCR amplification was conducted using 10 μL QIAGEN hot start master mix plus, 0.5 μM forward primer, 0.5 μM reverse primer, 6 μL H2O and 2 μL template. PCR reactions were as follows: 95°C for 5 min, followed by 40 cycles of denaturation at 95°C for 30s, annealing at 54°C for 30s and extension at 72°C for 70s followed by an extension cycle (72°C, 7 min). PCR products were viewed by electrophoresis (1% agarose gel) to confirm successful amplification of the functional gene and determine the presence or absence of the pseudogene. All samples initially indicating absence of the pseudogene were retested to confirm its absence. PCR products were cleaned with ExoSap IT (5 μL PCR product, 2 μL ExoSAP‐IT Express) and quantified by Qubit. Samples were submitted to the Iowa State University DNA Facility for sequencing. The functional gene was sequenced in both directions. The pseudogene was sequenced using the forward primer only. Resulting sequences were aligned to each other and white‐tailed deer PRNP sequences found in the NCBI database in MEGA 7. PRNP sequences found in the NCBI database were used as references from which to resolve the genotype at codon 96. The genotypes at codon 96 determined by the SNP array were compared to those determined by Sanger sequencing to identify agreement or mismatch between the two methods.

Differences in allele frequency of disease‐associated loci can lead to differences in disease risk and prevalence across various genetic clusters (Myles et al. 2008). To determine whether the minor allele frequencies of CWD‐associated loci in captive white‐tailed deer are comparable to those in free‐ranging white‐tailed deer that are not detected with CWD, we analysed the absolute differences between the 55 CWD‐associated minor alleles reported by Seabury et al. (2020) and the respective alleles in the OVSNP60.

## 3 Population Genetic Diversity and Structure Analysis

Genetic diversity summary statistics for the OVSNP600 and OVSNP60 arrays were calculated globally and for each state using the R packages snpR (*calc_* functions; v 1.2.11; Hemstrom and Jones 2023), adegenet (for file manipulation; v 2.1.11; Jombart 2008), dartR (*gl.report* functions; v 2.9.9.5; Gruber et al. 2018), and PopGenReport (function *allel.rich*; v 3.1; Adamack and Gruber 2014). Due to uneven sample sizes amongst states, allelic richness was estimated using rarefaction (Van Loon et al. 2007). Observed heterozygosity and unbiased expected heterozygosity were estimated and compared within each population using *F*_IS_ (Weir and Cockerham 1984; Nei 1978). Genetic differentiation between states was estimated by *F*_ST_ (Weir and Cockerham 1984) and plotted as a heatmap to visualise pairwise differences.

We compared the discriminatory power of the OVSNP600 and OVSNP60 arrays for identifying familial relationships by calculating the probability of identity amongst unrelated individuals (P_ID_) and amongst siblings (P_ID(sibs)_) for increasing locus combinations using the R package PopGenUtils (v 0.1.8, Tourvas 2021).

To explore the performance of the OVSNP600 and OVSNP60 arrays, patterns of population genetic structure were described by principal component analysis (PCA) using PLINK 1.9 (v1.90; Purcell et al. 2007). We filtered out loci with high linkage disequilibrium (indep‐pairwise 50 10 0.8). For each dataset, the first two principal components (PCs) were plotted using Program R (v4.1.2; R Core Team 2021) package ggplot2 (v 3.4.2; Wickham 2016) with samples colour‐coded by state of origin. In the PCA of the OVSNP60 samples, the Michigan samples formed two distinct clusters and prompted us to separate the samples at the natural geographical break between the Upper (UP) and Lower (LP) Michigan peninsulas. A second PCA was generated using the OVSNP60 dataset with the UP and LP Michigan samples only, to evaluate the capacity of the OVSNP60 to recover geographically based differentiation within states, despite the fact that our sample collection was not specifically designed to quantify spatial genetic structure at this scale.

Model‐based clustering was performed in ADMIXTURE (v 1.3.0; Alexander et al. 2009), and RStudio, based on an admixture ancestry model and correlation between allele frequencies. The potential number of genetic clusters (K) was estimated with ADMIXTURE using values of K based on the number of states with samples for each array. We evaluated K of 1 to 18 of the OVSNP600 and from 1 to 20 of the OVSNP60 at 10 CV‐folds for each K. The likelihood values for each repetition at each putative K were reported, and the optimal K value for each dataset was chosen as the one with the lowest cross‐validation error. Each sample's genome was proportionally assigned into these K ancestral clusters based on its allele frequency distribution. Population admixture bar plots, based on ADMIXTURE output, were plotted via R package ggplot2.

## 4 Results

### 4.1 Resequencing Variation

We generated whole‐genome resequencing data for 45 wild deer from Iowa, North Carolina, and Texas, resulting in an initial discovery of 89 million putative SNPs (BioProject accession: PRJNA870086). After two rounds of filtering, we retained a final set of 57,950,917 SNPs, out of which SNPs were selected for the development of the OVSNP600. One individual yielded coverage depth of only 3.3% and 36.8% of data were missing; this sample was excluded from downstream analyses. The depth of coverage for the 44 retained individuals ranged between 6.8 and 17.6, with relatively low proportions (0.5%–1.4%) of missing data (Table S2). The ratio of heterozygous to homozygous SNPs ranged from 1.41 to 2.15 and the transition to transversion ratio ranged from 2.43 to 2.46.

### 4.2 OVSNP600 Summary Data

**FIGURE 1:**
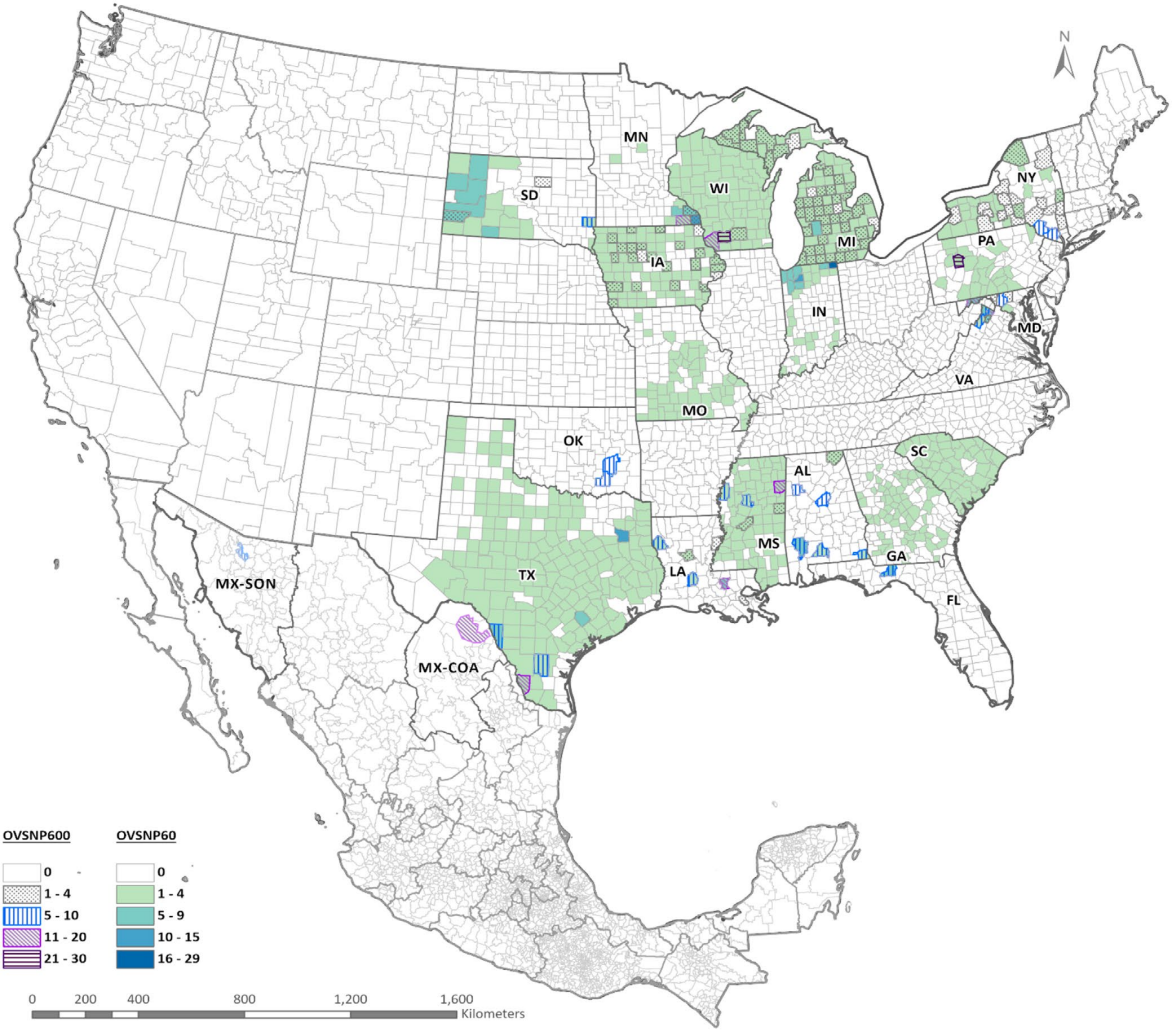
Sample distribution map for the OVSNP60 and the OVSNP600 dataset. Sample location data provided by sample collectors is mapped to state and county, with shading to indicate the sampling density within a county.

From the ~58 million SNPs identified through whole‐genome resequencing, probe sets for 702,183 SNPs were included on the Axiom OVSNP600 Genotyping Array. ThermoFisher uses DQC and QC call rate to determine which samples move on to genotyping. Two samples, one from IA and the other from MS, did not pass sample QC metrics. The remaining 478 total samples were processed and 469 (97.7%) were successfully genotyped and passed quality control standards, representing all 15 U.S. states and Mexico (Table S1, Figure 1). Sex distribution in the final OVSNP600 genotyped dataset varied by state, with a total of 142 (30.3%) females, 134 (28.6%) males, and 193 (41.1%) unrecorded (Table S3). Due to variations amongst data collection methods, age classes strongly differed amongst states; samples from the final OVSNP600 dataset were subsequently classified into 4 age classes (Table S3), including 21 (4.5%) fawns (<12 months of age), 28 (6.0%) yearlings (12–24 months of age), 240 (51.2%) adults (> 24 months of age), and 180 (38.4%) with an unknown age. Of the 702,183 SNPs on the OVSNP600, 517,261 (73.67%) spread across 7876 scaffolds, were categorised as ‘Best and Recommended’ by ThermoFisher, and were retained for further analyses and the development of the OVSNP60. The call rate of these ‘Best and Recommended’ SNPs ranged between 97.02% and 100% and included 4910 SNPs that were monomorphic for our set of samples.

### 4.3 OVSNP60 Summary Data

From the 517,261 ‘Best and Recommended’ SNPs identified through the OVSNP600, probe sets for 72,723 SNPs were included on the Axiom OVSNP60 Genotyping Array. Four samples from GA did not pass sample QC standards. The remaining 1529 samples were processed and 1392 (91%) were successfully genotyped and passed QC standards. Of the 1462 unique individuals included on the array from the 19 states, 1335 (91.3%) were successfully genotyped and represented 18 states (the single sample from Ohio that was included was not successfully genotyped; Table S1, Figure 1). The distribution of sex and age from samples included on the OVSNP60 varied by state; in total there were 677 males (50.7%), 409 females (30.6%), and 249 individuals with no sex data recorded (18.6%). Similarly to the OVSNP600, age classes were classified into 4 subgroups: fawns (*n* = 30; 2.3%), yearlings (*n* = 151; 11.3%), adults (*n* = 772; 58.1%) and unknown age (*n* = 377; 28.3%) (Table S3). Of the 72,723 SNPs on the OVSNP60, 64,839 SNPs (89.2%) spread across 6703 scaffolds were categorised as ‘Best and Recommended’ by ThermoFisher and were kept for further analyses. The call rate of these SNPs ranged between 97% and 100% and included one monomorphic SNP for our set of samples. Of the duplicate samples included for quality control, 51 of the OVSNP60 duplicates were genotyped with an overall error rate of 0.39% and four of the OVSNP600 duplicates were genotyped with an overall error rate of 0.48%. No duplicate individual had an error rate of more than 2%, and multilocus genotypes showed that duplicated samples were located in the same PCA space (Figure S3). Although only a small number of individuals (*n* = 5) were genotyped on both arrays, genotypes were highly concordant across panels, supporting the technical reliability of the assays. Broader replication in an independent study (Christensen et al. 2025) similarly found strong concordance between arrays, reinforcing their suitability and consistency for population genetic analysis. Nonetheless, the limited overlap constrains our ability to fully evaluate the comparability of population‐level metrics between the two panels. Broader replication would provide a more rigorous assessment of panel performance for population genetic applications.

Markers were selected for the array based on technical metrics such as probe performance and mutation type, rather than based on population‐level informativeness. We recognise that this strategy may influence the resolution of genetic structure and diversity estimates. Panels optimised for informativeness (e.g., Rosenberg et al. 2003; Rosenberg 2005; Biddanda et al. 2020) often produce stronger clustering and lower PIDs, whereas our technically driven selection may yield more conservative estimates of genetic diversity but supports broader applicability across management and population genetics applications. These outcomes reflect a tradeoff between maximising technical performance and achieving high informativeness, and they underscore the value of future evaluations using targeted marker selection approaches.

### 4.4 CWD Associated SNPs

All 15 probe sets on the OVSNP600 targeting codon 96 of the *PRNP* gene yielded genotypes; however, only 2 fell into the ‘Best and Recommended’ category. Of the 62 probesets that were included on the OVSNP60 targeting *PRNP* SNPs, only 1 probeset for each *PRNP* SNP (codons 95, 96, and AA116) and 45 of the 55 SNPs putatively associated with CWD in captive deer (Seabury et al. 2020) fell into the ‘Best and Recommended’ category.

Sanger sequencing results for the *PRNP* functional gene were obtained for 89 out of 96 tested samples and were compared to the corresponding genotype results from the OVSNP60 PRNP ellipse created by ThermoFisher (Figure S1). For samples identified within or above the codon 96 AA genotyping ellipse (*n* = 8) or nested between the AG and AA array genotyping ellipses (*n* = 20), all were confirmed by sequencing as genotype AA in the functional *PRNP* gene. For samples within or above the AG ellipse (*n* = 42), 39 were sequenced as heterozygous at the functional gene (AG) and 3 as GG. For samples that fell within or above the GG ellipse, 17 were sequenced as GG and 1 was AG. Sanger sequencing results for the *PRNPy* pseudogene revealed that 71 of the 89 samples which yielded successful functional gene sequences also had the *PRNPy* pseudogene. All pseudogene results produced sequences with the alternate homozygous genotype (GG). None of the samples within or above the AA ellipse (*n* = 8) had a pseudogene. All samples nested between the AG and AA ellipses (*n* = 20) had a pseudogene. For samples within or above AG ellipse (*n* = 42), 36 of the 39 heterozygous (AG) and all 3 of the homozygous (GG) samples had a pseudogene. For samples that fell within or above GG ellipse, 15 of the 17 homozygous (GG) had a pseudogene and the heterozygous sample (AG) did not have a pseudogene.

**FIGURE 2:**
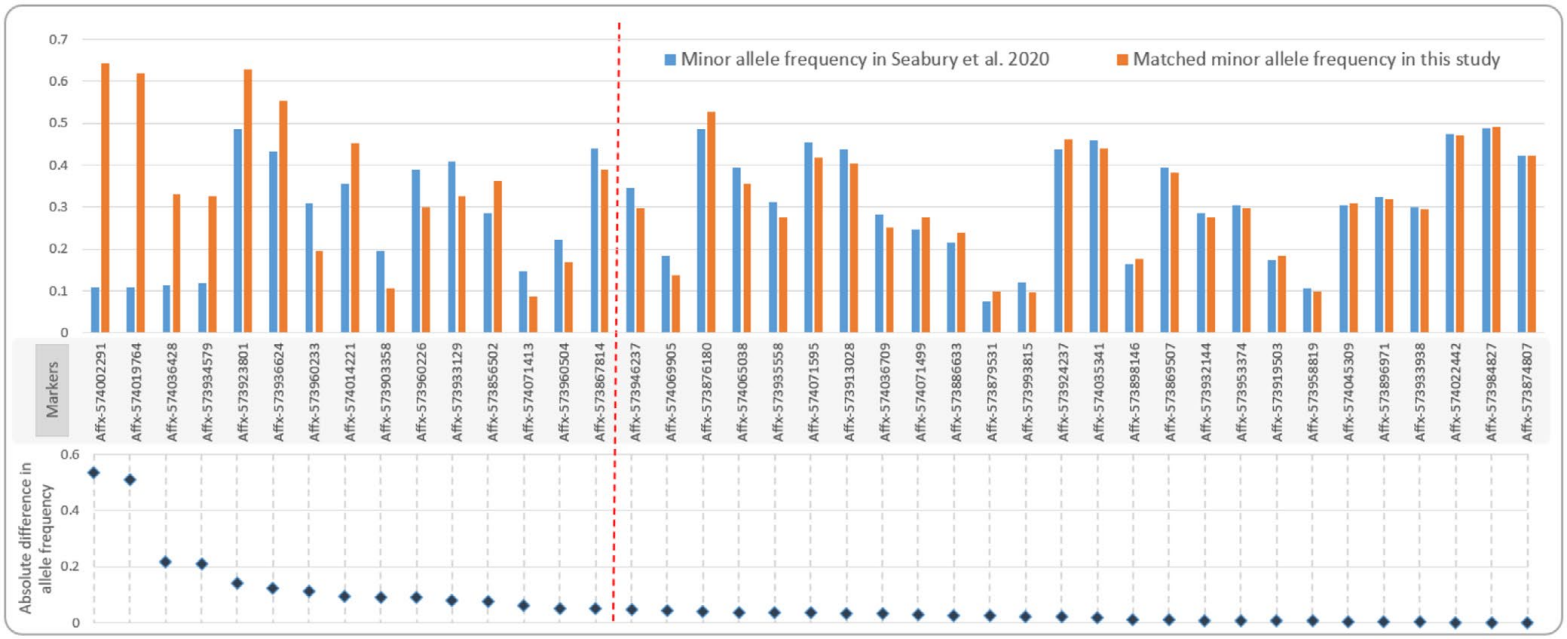
Comparison of allele frequencies at putative CWD‐associated loci. Minor allele frequencies for 41 loci associated with CWD status in the Seabury et al. (2020) dataset (blue bars) and their corresponding frequency in this study (orange bars). Loci are ordered by the difference in allele frequencies between the two studies; loci to the left of the red vertical dashed line have an absolute difference in allele frequency greater than 0.05.

Of the 55 unique CWD‐associated markers from Seabury et al. 2020 included in the OVSNP60, 41 markers fell into the ‘Best and Recommended’ category and were successfully genotyped in our dataset. Of the 41 loci retained, 36 had matching minor alleles in OVSNP60. The absolute difference between the test alleles of CWD‐associated loci and the corresponding alleles in OVSNP60 revealed a discrepancy, with 15 loci showing more than 0.05 difference (Figure 2).

### 4.5 Population Genetic Diversity and Structure

Despite varied sample collection strategies across states and arrays, genetic diversity estimates were nearly identical across arrays and broadly similar amongst states. The average allelic richness was close to 1.3 in all states for both arrays. Heterozygosity averaged around 0.31 (range 0.225–0.318 for the OVSNP600, 0.274–0.327 for the OVSNP60) with a slight deficiency of observed heterozygotes overall and in all states (Tables S4 and S5). Samples from Mexico, which were only represented in the OVSNP600, exhibited the lowest genetic diversity. This pattern could be due to small sample size; however, other states with small sample sizes in the OVSNP600 dataset had higher levels of genetic variation (e.g., FL and SD). It is also possible that the low genetic diversity we observed in Mexico was an artefact of ascertainment bias from our initial resequencing sample, which included samples from wide‐ranging populations in Texas, North Carolina, and Iowa specifically to minimise such bias.

Both arrays yielded extremely low P_ID_ and P_ID(sibs)_ indicating that even first order relatives (parent‐offspring or full siblings) are unlikely to share a multilocus genotype. The number of loci required to attain a P_ID_ of less than 0.0001 was 134 loci (or 223 loci for P_ID(sibs)_ < 0.0001) for the OVSNP60 (Figure S4). The number of loci required to achieve P_ID_ and P_ID(sibs)_ of less than 0.0001 for the OVSNP600 was much higher (6460 loci for PID < 0.0001 and 7130 loci for P_ID(sibs)_ < 0.0001; Figure S4), likely attributed to the locus selection process for the OVSNP600. The OVSNP600 included Axiom conversion types such as Mono High Resolution and No Minor Homozygote in OVSNP600 locus selection that were excluded in the OVSNP60, loci that would have little or no impact on P_ID_. For both arrays, a robust P_ID_ sufficient for assigning parentage was attained with fewer than 1% of genotyped loci.

Genetic differentiation was highest between Mexico and other states in the OVSNP600. Florida also exhibited high pairwise *F*_ST_ estimates (Figure S2). These patterns were likely driven in part by small sample sizes, but probably also because these populations are somewhat isolated. The larger number of samples in the OVSNP60 revealed stronger regional patterns of genetic differentiation. Florida was differentiated from all other states, likely due in part to small sample sizes from these states (Figure S2). However, other states with small sample sizes (e.g., Maryland) did not show similarly strong differentiation from other states, suggesting that the differentiation we observed in Florida is influenced by isolation. South Dakota exhibited differentiation from most other states; samples were taken mostly from the western half of the state and thus are more geographically separated from the rest of the OVSNP60 samples. The South Dakota samples are least differentiated from those that are geographically nearest, IA, MO and MN. South Carolina also stands out as differentiated from most other states, with differentiation patterns that are correlated with geography. For example, SC is most similar to nearby GA, AL, MS, and mid‐Atlantic states PA, NY and MD, and most differentiated from distant midwestern states IA, IN, MN, MO, SD and WI.

**FIGURE 3:**
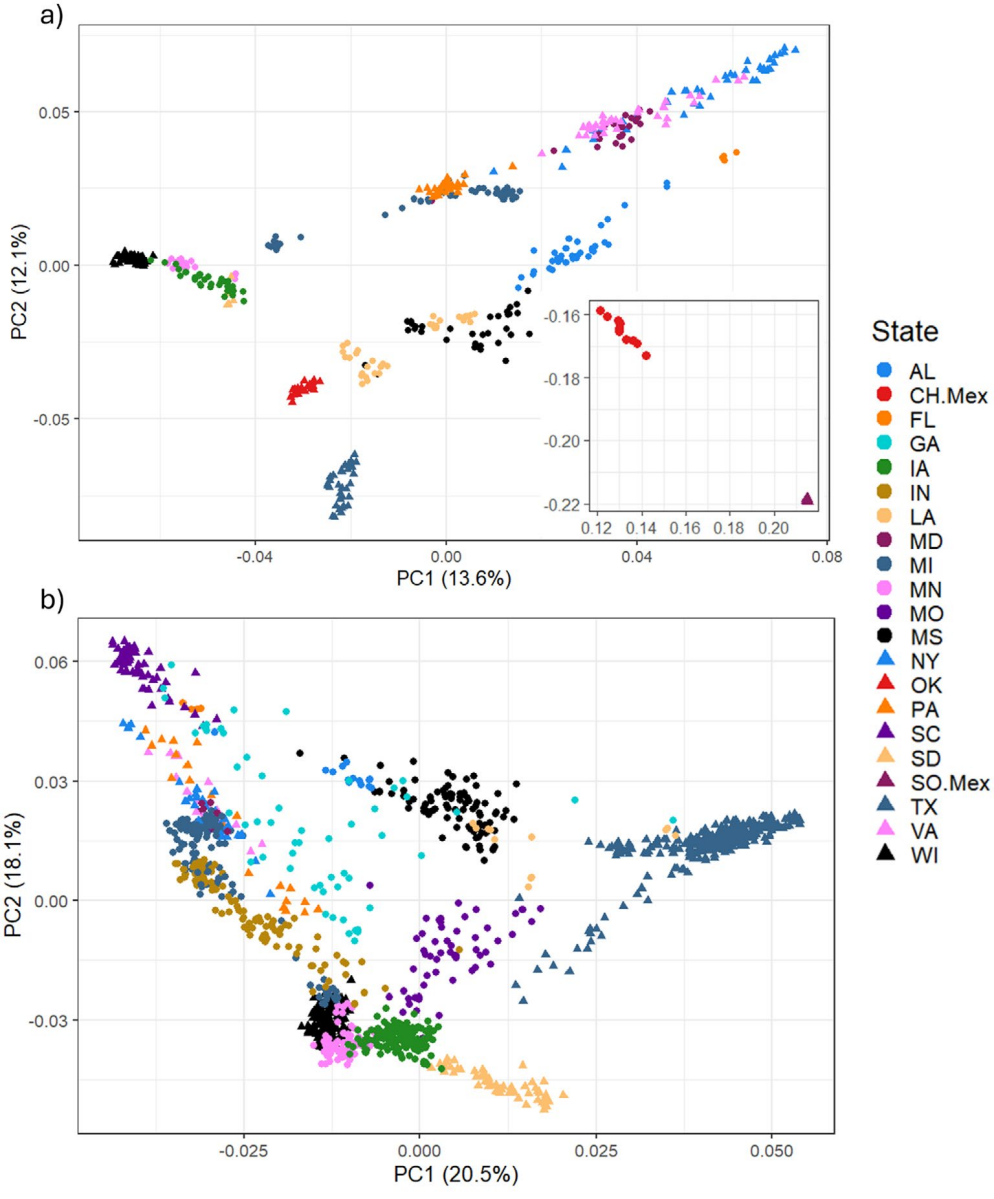
PCA analysis of the OVSNP60 and OVSNP600 genotypes. (a) Principal components analysis showing PC1 and PC2 for 469 individual deer sampled from 17 states in the US and Mexico and genotyped at 517,261 SNPs on the OVSNP600 array. Mexico samples from analysis provided in subplot for easier readability. (b) Principal components analysis showing PC1 and PC2 for 1335 individual deer sampled from 18 US states and genotyped at 64,839 SNPs on the OVSNP60 array. Samples are colour‐coded by state of origin.

The PCAs of both the OVSNP600 and OVSNP60 clearly separated the samples by their geographic origin (Figure 3). Initial pruning of the OVSNP600 data via LD Threshold removed 29,506 SNPs, leaving 487,775 for analysis. Using PCA for the OVSNP600 samples (*n* = 469), we calculated 20 eigenvalues, and revealed that the PC1 accounted for 13.6% of all the variation described in the OVSNP600, while PC2 accounted for 12.1% of all variation. PC1 indicates a distinction between the “eastern” (e.g., VA, AL, NY) and the “central” (e.g., IA, OK, SD) states (Figure 3), while PC2 highlights a potential separation between “northern” (e.g., WI, MN, PA) and “southern” (e.g., TX, LA, MX) states. This pattern was particularly distinct in the states of TX, OK, AL, FL, WI and both states in Mexico. Moreover, some overlap between clusters was also observed between certain states (e.g., MS and LA; IA, MN, and SD), all of which are close in geographic distance. Overall, the OVSNP600 PCA revealed five main clusters; one consisting of the samples from both states in Mexico, one made up of all TX samples, one of southern U.S. states (OK, LA, MS, AL and FL), one consisting of upper midwestern U.S. states (SD, MN, IA, WI and MI), and one of eastern U.S. states (MI, PA, NY, VA and MD). Michigan samples were split between the midwestern U.S. and eastern U.S. clusters.

Initial pruning of the OVSNP60 data via LD Threshold removed 556 SNPs, leaving 64,283 SNPs for further analyses. Using PCA for the OVSNP60 samples (*n* = 1335), we once again calculated 20 eigenvalues, and included the PC1 accounting for 20.5% of all the variation found in the OVSNP60, and the PC2 accounting for 18.1% of all the variation (Figure 3). While the PCA separated the OVSNP60 samples into clusters based on their geographic origin, more overlap between samples was observed, when compared to the OVSNP600 (Figure 3). The PCA showed at least seven main clusters, one consisting of TX samples, one of GA samples, one made up of southern U.S. states (AL, LA, and MS), two different clusters of midwestern U.S. states (IA, MI, MN, SD,and WI) and (IN, MI, NY, PA and VA), and two clusters of southeastern U.S. states (AL, LA and MS) and (FL and SC) in the OVSNP60 (Figure 3). Similar to the OVSNP600 the MI samples were split between two clusters.

Initial pruning of the MI dataset via LD Threshold removed 707 SNPs, leaving 64,132 for the final analysis. As before, we calculated 20 eigenvalues using PCA for the Michigan samples (*n* = 145). The first principal component (PC1), which accounted for 10.8% of all the variation in the dataset, and PC2, which accounted for 8.05% of all the variation of the data, highlighted a separation of the samples between the UP and the LP (Figure S5). Noteworthy, the OVSNP60 PCA showed that the MI samples from the UP clearly clustered with the WI samples, while the MI samples from the LP were closely clustered with the IN samples (Figure 3).

In‐depth clustering was captured by ADMIXTURE analysis of both the OVSNP600 and OVSNP60 datasets, which assumed that there were K populations characterised by a set of allele frequencies at each SNP. To determine the most likely number of populations, the posterior probability was calculated for each value of K based on the estimated log posterior value of K and individual assignment patterns. Cross‐validation results of K for K = 1–11 for the OVSNP600 revealed that K = 5 was the most likely with the lowest cross‐validation error value at 0.562 (Figure S6), while the lowest cross‐validation error of K = 1–20 for the OVSNP60 was 0.564 at K = 11 (Figure S6). Cross‐validation errors were similarly low for K values around these optimal Ks, likely reflecting the overlap amongst clusters especially in the OVSNP60, similar to what we observed in the PCAs.

At K = 5 for the OVSNP600 samples, some states were clearly assigned to a subpopulation but many other states had a mixture of SNPs from different subpopulations (Figure S7). Individuals from WI, IA, SD, and MN were assigned to S1; VA, NY, and MD, PA were assigned to S2; OK and TX were assigned to S3; samples from Mexico were assigned to S4; AL, FL, LA, and MS were assigned to S5. Individuals from Michigan had a mixed proportion of SNPs from S1 and S2, which could be a result of deer being from the UP and LP. These results supported the patterns found in our PCA analysis and also showed that a higher proportion of SNPs was shared between states in closer geographic proximity.

At K = 11 for the OVSNP60 samples, most states had a dominant proportion of SNPs from one genetic subpopulation (Figure S8). Overall, most states can be differentiated from one another based on their SNP composition; for example, SD was primarily assigned to S10, TX to S6, and MN to S11. We also observed some instances where adjacent states shared similar assignment patterns, such as in the southeast (SC, FL), Gulf States (AL, LA, MS), and mid‐Atlantic (MD, NY, PA, and VA). Clear assignment patterns for most states, with some degree of overlap indicating shared variation amongst geographically adjacent states, were similar to what we observed in the PCA analysis.

## 5 Discussion

In this study, we developed high (OVSNP600; ~700,000 SNPs) and medium (OVSNP60; ~70,000 SNPs) density SNP arrays as two novel high‐throughput genomic resources to improve monitoring and management of white‐tailed deer populations. Both arrays exhibited high genotyping success across individuals and SNPs and produced high‐quality genotypes with low error rates, demonstrating their efficiency and quality. To make these tools accessible to other laboratories and to address interstate management objectives, it was key to survey genetic diversity broadly enough to make these arrays useful across states without sacrificing genotyping success. Additionally, the panels are not targeted toward any particular aim and thus provide accurate estimates of genome‐wide diversity that are robust to biases associated with marker selection to maximise informativeness for specific research goals. Although our sampling was concentrated in the white‐tailed deer's eastern and central range, the SNPs included on both arrays performed well across all surveyed states. In addition to broadly surveying genetic variation, these arrays can also be used to survey variation at specific loci relevant to management. In particular, the genotyping success of the CWD‐associated SNPs across all samples shows potential for using these arrays to inform CWD management of wild populations of white‐tailed deer.

Given the difficulty in diagnosing CWD in living animals, long incubation times, a lack of vaccination tools or therapeutic treatments, and because deer are highly mobile, genomic tools can be especially important for understanding host contact networks, disease transmission dynamics, and risk landscapes. Polymorphisms in the *PRNP* gene are linked to prevalence and disease progression rate in infected individuals (O'Rourke et al. 2004; Johnson et al. 2006, Johnson et al. 2011; Robinson, Samuel, O'Rourke, and Johnson 2012; Robinson, Samuel, Lopez, and Shelton 2012; Denkers et al. 2020; Arifin 2021). We included 15 probesets within the *PRNP* gene on the OVSNP600, only two of which passed quality controls to be included on the array, and 62 PRNP probesets on the OVSNP60 with only three passing quality control filters. This low success rate for PRNP probesets is attributable to the presence of the *PRNPy* pseudogene, which is present in most but not all white‐tailed deer and exhibits high sequence similarity to the functional *PRNP* gene. Supplementary Sanger sequencing was required to genotype the functional *PRNP* in the presence of the *PRNPy* pseudogene. Most of the samples (80%) we sequenced contained the *PRNPy* pseudogene; this is much higher than what has been reported in other studies (O'Rourke et al. 2004; Kelly et al. 2008), though our goal here was not to survey *PRNPy* prevalence but rather to explore the clustering patterns of PRNP in the presence of *PRNPy*. Samples containing the *PRNPy* pseudogene were found within or above the GG and AG array genotyping ellipses at the codon 96 SNP, or between the AG and AA ellipses. They were not found within the AA ellipse, though we sequenced few samples from this ellipse. Future work to refine genotype calling for this locus, perhaps by adapting software designed for the detection of copy number variation (e.g., Wang et al. 2007; Karunarathne et al. 2023) and using a multi‐allelic genotyping approach (e.g., Handsaker et al. 2015; Danecek et al. 2021) would be useful for collecting *PRNP* genotypes for both the functional and pseudogene directly from the arrays.

Genome‐wide association studies in captive white‐tailed deer have shown an association between CWD presence and loci outside the *PRNP* gene (Seabury et al. 2020). We included all 55 of those loci in our arrays; 41 of them met the ‘Best and Recommended’ criteria in our sample of free‐ranging white‐tailed deer sequenced using OVSNP60. Thermo Fisher Scientific's policies permitted only CWD‐nondetected samples to be included in the array development dataset, thus we could not do a GWAS analysis in this study. Fifteen loci (37%) showed an allele frequency difference of more than 0.05, implying potential differences in CWD risk between these two datasets. Without CWD+ samples, the absolute difference in test/minor allele frequencies is of limited use to inform whether the loci associated with CWD in captive deer are also associated with CWD status in wild white‐tailed deer. However, the presence of these loci on both arrays offers exciting avenues for future research on CWD progression and risk assessment in wild white‐tailed deer.

The development of custom microarray panels that effectively survey genome‐wide variation provides tools for addressing key evolutionary and management topics at a variety of scales, such as genetic diversity and structure (Ba et al. 2017; Wang et al. 2017), individual identification and relatedness (Hayward et al. 2022; Kleinman‐Ruiz et al. 2017), and natal origin traceability (Norman and Spong 2015; Puckett and Eggert 2016). The abundance and distribution of markers across the genome, data accuracy and repeatability, increases in cost‐efficiency, and greater accessibility of bioinformatic pipelines offer SNP panels an advantage over other marker types such as microsatellites (Montgomery et al. 2005; Fernández et al. 2013; Lemopoulos et al. 2019; Zimmerman et al. 2020). Additionally, SNPs are typically better for genotyping low‐quality samples, including historical, noninvasively collected, or otherwise degraded samples (Morin and McCarthy 2007; Campbell and Narum 2009). The arrays we developed for white‐tailed deer yielded extremely low P_ID_ and P_ID(sibs)_ values, indicating that it is unlikely that any two white‐tailed deer, even if they are closely related, will share a multilocus genotype across these arrays. Thus, only a small number of loci from these arrays would be needed for parentage assignment or sibship reconstruction. Our data indicate that a few hundred of the loci on the arrays would be needed for accurate parentage assignment, even when deer are related (P_ID(sibs)_ < 0.0001). This is in line with what has been found for other species using SNPs for parentage analysis (summarised in Flanagan and Jones 2019), further attesting to the discriminatory power of both the OVSNP600 and OVSNP60.

The genome‐wide data generated from both arrays were sufficient to cluster samples by their state of origin, showing general patterns of geographically associated genetic differentiation. Samples from Mexico, included in the OVSNP600, were highly differentiated in the PCA from the rest of the deer genotyped. Amongst US states, genetic differentiation estimates and results from both PCA and admixture analyses from both the OVSNP600 and OVSNP60 arrays suggested that deer exhibit geographic genetic clustering, and that there is admixture between states sharing physical borders. This finding is readily explained by the wide distribution, movement behaviours, and dispersal capacities of white‐tailed deer. However, we also found some signatures of admixture between states that do not share physical borders. Particularly in the OVSNP60 dataset, PCA results suggested that deer from some states, such as Louisiana, may be genetically similar to more distant locations in Texas, Mississippi, and Missouri. Deer from Georgia were even less clustered in the PCA plot with a large number of samples from southeast, mid‐Atlantic, and midwestern states. Finally, some deer from Texas grouped more closely with deer of upper‐Midwest ancestry than Texas. These findings suggest ancestry in some regions could be the result of long‐distance natural dispersal or historical translocations (e.g., McVean 2009; Chafin et al. 2021; Budd et al. 2018). Following near extirpation of white‐tailed deer in the late 19th and early 20th century in some states, restoration relied on extensive translocations of deer from regions with healthy population abundance. In the south‐eastern US, restocking included deer from outside the region (e.g., Mexico, Wisconsin; Blackard 1971, Smith et al. 1976), leaving quantifiable genetic signatures of this mixed founder stock (Leberg et al. 1994; Leberg and Ellsworth 1999; DeYoung et al. 2003; Doerner et al. 2005). Our sampling was not designed to test specific hypotheses regarding the origin of potential translocations, but our results suggest that our SNP arrays might be useful in a forensic capacity by identifying long‐distance translocations as well as to understand the evolutionary legacy of documented and undocumented translocations.

The two arrays presented in this study are powerful and complementary tools to evaluate genome‐wide patterns in genetic diversity and structure at both broad and fine scales, paving the way for novel insights into adaptation and advances in management. Clustering was stronger in the OVSNP600, due to the larger number of loci in this array, but both arrays accurately surveyed genetic variation and showed similar patterns of genetic structure that are in line with what we know about white‐tailed deer biology. There is a tradeoff between the number of samples and the number of loci that can be incorporated into a research project. Increasing the sample size often results in greater gains in statistical power than increasing the number of loci, in particular given low SNP mutation rates and when *F*_ST_ is low as predicted for a highly mobile species (Kalinowski 2005; Morin et al. 2009). As such, running more samples on the OVSNP60, which has a lower per‐sample cost, might be a better strategy to maximise power for population genetic studies than genotyping more loci. In contrast, the larger number of loci on the OVSNP600 might be preferred when identifying loci associated with phenotypes or environmental features (Rellstab et al. 2015), or for inferring historical demography in a species with large population sizes and high levels of gene flow, and therefore small blocks of linkage disequilibrium (Lowry et al. 2017). Variation in species biology, historical demography, and genome evolution call for power simulations prior to starting any study to aid in optimising the tradeoff between the costs of sampling across the genome and across the landscape. While these arrays are robust and versatile genomic resources, conclusions should be interpreted in light of the tradeoffs inherent in their design and evaluation. Both arrays prioritised technical performance and broad applicability over population‐specific informativeness, and replication across panels was limited. Future work incorporating larger cross‐platform comparisons and alternative marker selection strategies could help refine tools for different applications.

Both of the SNP arrays presented in this study are valuable high‐throughput genomic resources for white‐tailed deer population management and for facilitating data sharing, large‐scale studies, and potentially long‐term genetic monitoring. The OVSNP600 and OVSNP60 arrays are commercially available and can be used to genotype a consistent marker set distributed across the white‐tailed deer reference genome. Future studies can apply these SNPs to help resolve questions about disease transmission, parentage and relatedness, detection of poaching or illegal translocations, and landscape genomics. Ready access to high‐density SNP arrays will also provide new opportunities to investigate cervid evolution, including taxonomic relationships and the effects of historical translocations (Sumners et al. 2015; Cohen et al. 2018). The OVSNP600 may be especially useful to identify candidate genes associated with disease susceptibility, local adaptation, or other phenotypes of interest. One of the biggest advantages of these SNP arrays is the increased repeatability and comparability of genotype data relative to more conventional approaches used in non‐model species, such as microsatellite analysis, and genotyping‐by‐sequencing. The development of genotyping arrays for model organisms and agricultural species, such as mice and cows has helped to rapidly advance our understanding of the genomic mechanisms behind disease susceptibility and prevention. Our aim is that developing similar tools for white‐tailed deer will help to increase access for molecular research on cervids to assist management throughout North America and facilitate the development of more targeted resources, such as genotyping‐by‐thousand sequencing (GT‐seq) panels (Campbell et al. 2015) to address specific management aims. These resources can provide a means of rapidly generating data that can be readily integrated and shared beyond political jurisdictions. This is important for a wide‐ranging, broadly distributed species like white‐tailed deer and will enhance the ability of researchers and managers challenged with understanding, managing, and mitigating the spread of CWD.

## Author Contributions

D.N. performed lab work and analysed data for both arrays and wrote the manuscript with help from all authors. J.A.B., E.K.L., C.N.O.‐C., R.W.D., D.P.W. designed the research for all parts of the study, helped with interpretation of data, and provided samples for the OVSNP600 array. A.K.T. and P.T.E. helped with data interpretation. C.R.G. helped with data interpretation and figures. W.A.L., A.S.S., A.J.S., J.M.R., and Z.‐L.H. analysed data for the deer resequencing dataset. J.R.C., M.C., J.N.C., C.H.K., M.L.L., W.T.M., A.S.N., K.L.S., D.J.S., J.A.S., and W.D.W. provided samples for the OVSNP60 array. All authors provided feedback on the article and have approved the final version.

## Conflicts of Interest

The authors declare no conflicts of interest.

## Supporting information


**Appendix S1:** men70085‐sup‐0001‐AppendixS1.docx.

## Data Availability

All programmes for analysis used by the authors are provided in the article. Resequencing data are deposited to NCBI Bioproject Database Accession: PRJNA870086. State‐level data are protected by individual state agencies and thus are not made public; the data may be available upon reasonable request from the corresponding author with appropriate permissions.
