# Supplementary material for: Development of High‐Throughput Genomic Resources to Inform White‐Tailed Deer Population and Disease Management
Source: Mol Ecol Resour. 2025 Nov 26;26(1):e70085. doi: 10.1111/1755-0998.70085 (PMC12648641; doi:10.1111/1755-0998.70085)

Supplementary Materials for:

**Development of High-Throughput Genomic Resources to Inform White-tailed Deer Population and Disease Management**

**Molecular Ecology Resources**

**Authors:** David Navarro^1^, Emily K. Latch^2^, Anaïs K. Tallon^2^, Caitlin N. Ott-Conn^3^, Randy W. DeYoung^4^, Daniel P. Walsh^5^, Peter T. Euclide^6^, Chandika RG^2^, Wes A. Larson^7^, Arun S. Seetharam^8^, Andrew J. Severin^8^, James M. Reecy^9^, Zhi-Liang Hu^9^, Jay Cantrell^10^, Michelle Carstensen^11^, Joe Caudell^12^, Charlie Killmaster^13^, Mitch Lockwood^14^, William McKinley^15^, Andrew Norton^16^, Krysten Schuler^17^, Daniel Storm^18^, Jason Sumners^19^, W. David Walter^20^, Julie A. Blanchong^1^

^1^ Department of Natural Resource Ecology and Management, Iowa State University, Ames, IA 50011, USA

^2^ Behavioral and Molecular Ecology Research Group, Department of Biological Sciences, University of Wisconsin-Milwaukee, Milwaukee, WI, 53211, USA

^3^ Michigan Department of Natural Resources

^4^ Caesar Kleberg Wildlife Research Institute, MSC 218, Texas A&M University-Kingsville, Kingsville, TX 78363, USA

^5^ U.S. Geological Survey, Montana Cooperative Wildlife Research Unit, University of Montana, Wildlife Biology Program, Missoula, MT 59812, USA

^6^ Department of Forestry and Natural Resources, Illinois-Indiana Sea Grant, Purdue University 47907, , https://orcid.org/0000-0002-1212-0435

^7^ Alaska Fisheries Science Center, NOAA Alaska Fisheries Science Center, Auke Bay Laboratories, Juneau, Alaska 99801 USA

^8^ Office of Biotechnology, Genome Informatics Facility, Iowa State University, Ames, Iowa, USA, 50011

^9^ Department of Animal Science, Iowa State University, Ames, Iowa, USA 50011

^10^ South Carolina Department of Natural Resources Big Game Program

^11^ Minnesota Department of Natural Resources, Wildlife Health Program, 5463 West Broadway, Forest Lake, Minnesota, 55025, USA

^12^ Indiana Department of Natural Resources

^13^ Georgia Department of Natural Resources

^14^ Texas Parks and Wildlife Department

^15^ Mississippi Department of Wildlife, Fisheries, and Parks

^16^ South Dakota Game, Fish & Parks

^17^ Department of Public & Ecosystem Health Cornell University College of Veterinary Medicine Ithaca, NY 14853

^18^ Wisconsin Department of Natural Resources, 1300 W. Clairemont Eau Claire, WI 54701

^19^ Missouri Department of Conservation, 2901 West Truman Blvd Jefferson City, MO 65109

^20^ U.S. Geological Survey, Pennsylvania Cooperative Fish and Wildlife Research Unit, The Pennsylvania State University, University Park, PA 16802

**Supplementary Table S1. Samples included on the OVSNP600 and OVSNP60 arrays.** Summary of the number of unique samples included on each array and the number of samples that met genotyping quality control thresholds for the OVSNP600 and OVSNP60 genotyping arrays.

| **State** | **# Samples Included on OVSNP600** | **# Samples Successfully Genotyped on OVSNP600** | **# Samples Included on OVSNP60** | **# Samples Successfully Genotyped on OVSNP60** |
| --- | --- | --- | --- | --- |
| **Alabama** | 34 | 34 | 14 | 14 |
| **Florida** | 5 | 5 | 5 | 4 |
| **Georgia** | - | - | 65 | 54 |
| **Indiana** | - | - | 107 | 96 |
| **Iowa** | 35 | 34 | 144 | 141 |
| **Louisiana** | 37 | 37 | 12 | 12 |
| **Maryland** | 26 | 26 | 7 | 7 |
| **Michigan** | 51 | 51 | 148 | 145 |
| **Minnesota** | 25 | 24 | 95 | 74 |
| **Mississippi** | 36 | 33 | 109 | 90 |
| **Missouri** | - | - | 65 | 56 |
| **New York** | 43 | 43 | 38 | 36 |
| **Ohio** | - | - | 1 | 0 |
| **Oklahoma** | 20 | 20 | - | - |
| **Pennsylvania** | 29 | 29 | 25 | 19 |
| **South Carolina** | - | - | 74 | 57 |
| **South Dakota** | 7 | 6 | 58 | 56 |
| **Texas** | 35 | 35 | 348 | 327 |
| **Vermont** | - | - | 0 | - |
| **Virginia** | 36 | 36 | 20 | 20 |
| **Wisconsin** | 43 | 43 | 127 | 123 |
| **Sonora, Mexico** | 8 | 3 | - | - |
| **Coahuila, Mexico** | 10 | 10 | - | - |
| **Totals** | **480** | **469** | **1462** | **1335** |

( - ) State did not provide samples for the array.

**Supplementary Table S2. Samples included on the OVSNP600 and OVSNP60 arrays.** Summary of total number of SNPs at each filtering step. Starting SNPs for the OVSNP600 were obtained from the resequencing data and staring SNPs for the OVSNP60 were obtained from the OVSNP600.

|  | OVSNP600 | OVSNP60 |
| --- | --- | --- |
| Total SNPs Starting SNPs | 5,242,424 | 517,261 |
| Selected SNPs for the Array | 702,182 | 72,723 |
| Thermofisher Best and Recommended SNPs | 517,261 | 64,839 |
| LD 0.8 Threshold Pruning | 487,775 | 64,283 |

**Supplementary Table S3.** **Genotyped samples by age and sex data for the OVSNP600 and OVSNP60 arrays.** Summary of the number of Males, Females, Fawns, Yearlings, and Adults from each state that met genotyping quality control steps for the OVSNP600 and OVSNP60 arrays. This metadata was provided by the collector of the samples and any missing data or unsubmitted data was labeled as unknown (Unk).

|  | **OVSNP600** | | | | | | | **OVSNP60** | | | | | | |
| --- | --- | --- | --- | --- | --- | --- | --- | --- | --- | --- | --- | --- | --- | --- |
| **State** | **Male** | **Female** | **Unk** | **Fawn** | **Yearling** | **Adult** | **Unk** | **Male** | **Female** | **Unk** | **Fawn** | **Yearling** | **Adult** | **Unk** |
| **AL** | 0 | 0 | 34 | 0 | 0 | 0 | 34 | 0 | 0 | 14 | 0 | 0 | 0 | 14 |
| **FL** | 0 | 0 | 5 | 0 | 0 | 0 | 5 | 0 | 0 | 4 | 0 | 0 | 0 | 0 |
| **GA** | - | - | - | - | - | - | - | 32 | 6 | 16 | 0 | 0 | 29 | 25 |
| **IA** | 14 | 20 | 0 | 0 | 0 | 34 | 0 | 104 | 38 | 0 | 0 | 45 | 97 | 0 |
| **IN** | - | - | - | - | - | - | - | 70 | 26 | 1 | 4 | 28 | 55 | 10 |
| **LA** | 0 | 0 | 37 | 0 | 0 | 0 | 37 | 0 | 0 | 12 | 0 | 0 | 0 | 12 |
| **MD** | 9 | 17 | 0 | 4 | 0 | 21 | 1 | 2 | 5 | 0 | 0 | 0 | 6 | 1 |
| **MI** | 27 | 24 | 0 | 0 | 20 | 31 | 0 | 71 | 74 | 0 | 10 | 43 | 92 | 0 |
| **MN** | 16 | 7 | 1 | 0 | 8 | 14 | 2 | 50 | 22 | 2 | 13 | 12 | 46 | 3 |
| **MO** | - | - | - | - | - | - | - | 0 | 0 | 56 | 0 | 0 | 0 | 56 |
| **MS** | 0 | 0 | 33 | 0 | 0 | 0 | 33 | 51 | 35 | 5 | 3 | 11 | 72 | 5 |
| **Coahuila, MX** | 0 | 0 | 10 | 0 | 0 | 0 | 10 | - | - | - | - | - | - | - |
| **Sonora, MX** | 0 | 0 | 3 | 0 | 0 | 0 | 3 | - | - | - | - | - | - | - |
| **NY** | 23 | 20 | 0 | 0 | 0 | 43 | 0 | 18 | 16 | 0 | 0 | 3 | 3 | 28 |
| **OH** | - | - | - | - | - | - | - | 0 | 0 | 0 | 0 | 0 | 0 | 0 |
| **OK** | 0 | 0 | 20 | 0 | 0 | 0 | 20 | - | - | - | - | - | - | - |
| **PA** | 12 | 17 | 0 | 0 | 0 | 29 | 0 | 9 | 12 | 0 | 0 | 0 | 4 | 17 |
| **SC** | - | - | - | - | - | - | - | 29 | 28 | 0 | 0 | 6 | 51 | 0 |
| **SD** | 4 | 2 | 0 | 1 | 0 | 5 | 0 | 31 | 24 | 1 | 0 | 0 | 0 | 56 |
| **TX** | 0 | 0 | 35 | 0 | 0 | 0 | 35 | 200 | 115 | 12 | 0 | 0 | 310 | 17 |
| **VA** | 16 | 20 | 0 | 0 | 0 | 36 | 0 | 10 | 8 | 2 | 0 | 3 | 8 | 9 |
| **WI** | 13 | 15 | 15 | 16 | 0 | 27 | 0 | 0 | 0 | 124 | 0 | 0 | 0 | 124 |
| **Total** | 134 | 142 | 193 | 21 | 28 | 240 | 180 | 677 | 409 | 249 | 30 | 151 | 773 | 381 |

( - ) State did not provide samples for the array.

**Supplementary Table S4. OVSNP600 genetic diversity table.** Sample size (n), allelic richness (Ar), nucleotide diversity (pi), observed heterozygosity (Ho), unbiased expected heterozygosity (uHe), gene diversity (Hs), and inbreeding coefficient (FIS) for each state and overall.

| **State** | **n** | **Ar** | **p_i_** | **Ho** | **uHe** | **Hs** | **F_IS_** |
| --- | --- | --- | --- | --- | --- | --- | --- |
| AL | 34 | 1.31 | 0.306 | 0.284 | 0.306 | 0.973 | 0.069 |
| FL | 5 | 1.27 | 0.267 | 0.262 | 0.267 | 0.900 | 0.017 |
| IA | 34 | 1.32 | 0.319 | 0.307 | 0.318 | 1.055 | 0.035 |
| LA | 37 | 1.31 | 0.313 | 0.287 | 0.313 | 0.985 | 0.079 |
| MD | 26 | 1.31 | 0.305 | 0.292 | 0.305 | 1.001 | 0.043 |
| MI | 51 | 1.31 | 0.312 | 0.294 | 0.312 | 1.009 | 0.057 |
| MN | 24 | 1.31 | 0.315 | 0.306 | 0.315 | 1.050 | 0.028 |
| MS | 33 | 1.31 | 0.313 | 0.291 | 0.313 | 0.998 | 0.066 |
| MX | 13 | 1.23 | 0.225 | 0.209 | 0.225 | 0.717 | 0.058 |
| NY | 43 | 1.30 | 0.300 | 0.285 | 0.300 | 0.977 | 0.050 |
| OK | 20 | 1.31 | 0.310 | 0.292 | 0.310 | 1.003 | 0.052 |
| PA | 29 | 1.31 | 0.311 | 0.302 | 0.311 | 1.036 | 0.030 |
| SD | 6 | 1.32 | 0.315 | 0.308 | 0.315 | 1.056 | 0.021 |
| TX | 35 | 1.31 | 0.315 | 0.298 | 0.315 | 1.024 | 0.051 |
| VA | 36 | 1.30 | 0.304 | 0.289 | 0.304 | 0.992 | 0.047 |
| WI | 43 | 1.31 | 0.309 | 0.299 | 0.309 | 1.025 | 0.033 |
| **Overall** | 469 | 1.30 | 0.302 | 0.291 | 0.318 | 0.988 | 0.053 |

**Supplementary Table S5.** **OVSNP60 genetic diversity table.** Sample size (n), allelic richness (Ar), nucleotide diversity (pi), observed heterozygosity (Ho), unbiased expected heterozygosity (uHe), gene diversity (Hs), and inbreeding coefficient (FIS) for each state and overall.

| **State** | **n** | **Ar** | **p_i_** | **Ho** | **uHe** | **Hs** | **F_IS_** |
| --- | --- | --- | --- | --- | --- | --- | --- |
| AL | 14 | 1.31 | 0.311 | 0.287 | 0.311 | 0.943 | 0.069 |
| FL | 4 | 1.26 | 0.274 | 0.271 | 0.274 | 0.891 | 0.009 |
| GA | 54 | 1.32 | 0.320 | 0.299 | 0.321 | 0.983 | 0.065 |
| IA | 142 | 1.33 | 0.327 | 0.315 | 0.327 | 1.036 | 0.036 |
| IN | 97 | 1.32 | 0.319 | 0.304 | 0.319 | 0.999 | 0.047 |
| LA | 12 | 1.31 | 0.319 | 0.297 | 0.319 | 0.975 | 0.063 |
| MD | 7 | 1.30 | 0.313 | 0.302 | 0.313 | 0.991 | 0.031 |
| MI | 145 | 1.32 | 0.319 | 0.301 | 0.319 | 0.988 | 0.056 |
| MN | 74 | 1.32 | 0.323 | 0.314 | 0.323 | 1.033 | 0.027 |
| MO | 56 | 1.32 | 0.326 | 0.311 | 0.326 | 1.021 | 0.045 |
| MS | 91 | 1.32 | 0.324 | 0.302 | 0.324 | 0.992 | 0.067 |
| NY | 34 | 1.31 | 0.314 | 0.301 | 0.314 | 0.988 | 0.039 |
| PA | 21 | 1.31 | 0.316 | 0.299 | 0.316 | 0.984 | 0.047 |
| SC | 57 | 1.30 | 0.301 | 0.281 | 0.301 | 0.924 | 0.062 |
| SD | 56 | 1.31 | 0.311 | 0.301 | 0.312 | 0.989 | 0.033 |
| TX | 327 | 1.33 | 0.326 | 0.305 | 0.326 | 1.002 | 0.064 |
| VA | 20 | 1.31 | 0.310 | 0.296 | 0.311 | 0.974 | 0.041 |
| WI | 124 | 1.32 | 0.323 | 0.309 | 0.323 | 1.014 | 0.045 |
| **Overall** | 1335 | 1.31 | 0.315 | 0.300 | 0.316 | 0.985 | 0.052 |

**Supplementary Figure S1. Genotype results for Codon 96 PRNP** **SNP.** Genotype plot for the Codon 96 PRNP SNP created by Thermofisher using the Axiom Analysis Software. Individual genotyping results were color coded based on the genotype group it most likely belonged to (AA, AB, or BB).

**
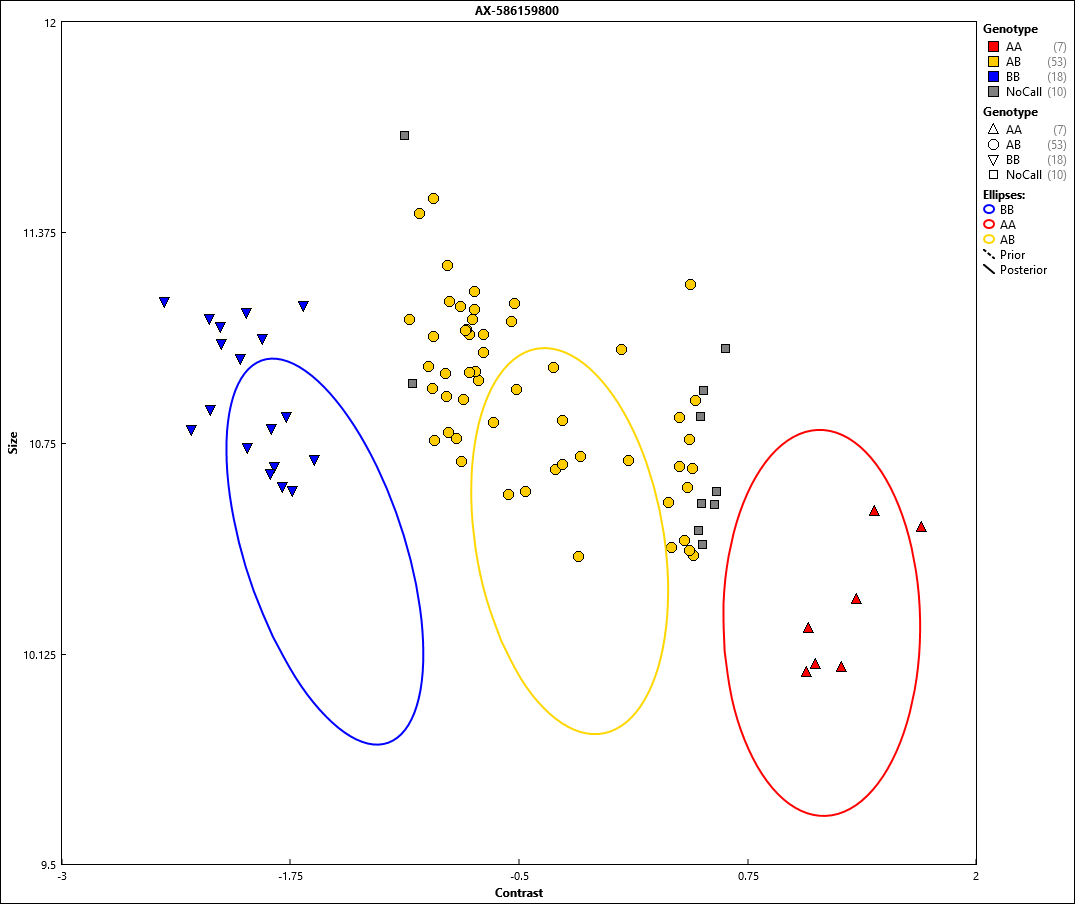
**

**Supplementary Figure S2. Pairwise FST for the OVSNP600 (a) and OVSNP60 (b).** FST analysis for 469 individual deer sampled from 17 states in the US and Mexico and genotyped at 517,261 SNPs on the OVSNP600 array (a) and for 1,335 deer sampled from 18 states genotyped at 64,839 SNPs on the OVSNP60 array (b).

(a) **OVSNP600** (b) **OVSNP60**
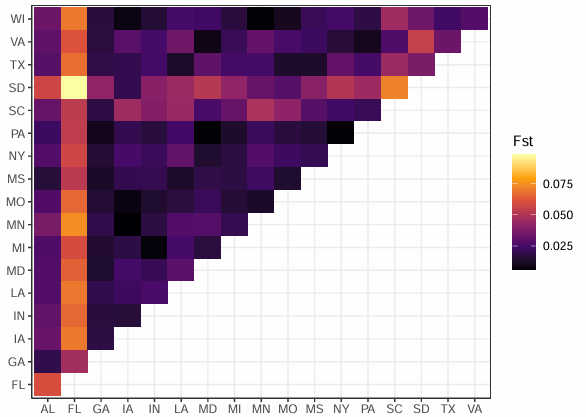

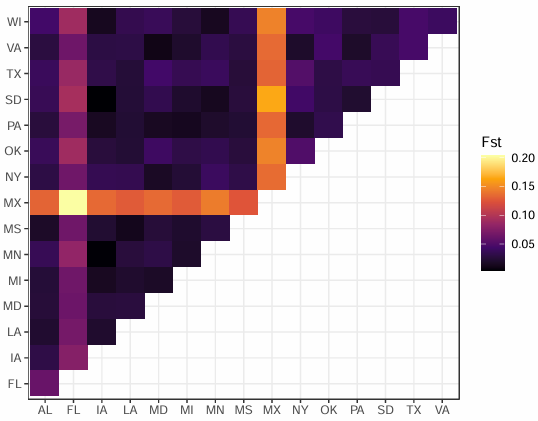


**Supplementary Figure S3. PCA showing overlap of duplicated samples from OVSNP600 and OVSNP60 arrays.** Principal components analysis showing PC1 and PC2 for 4 samples genotyped at 517,261 SNPs on the OVSNP600 array and 64,132 SNPs on the OVSNP60 array. Genotypes from the OVSNP600 array were trimmed to match the results of the OVSNP60 array before comparison of results. Duplicate samples are denoted by color; OVSNP600 results are circles and OVSNP60 results are triangles.


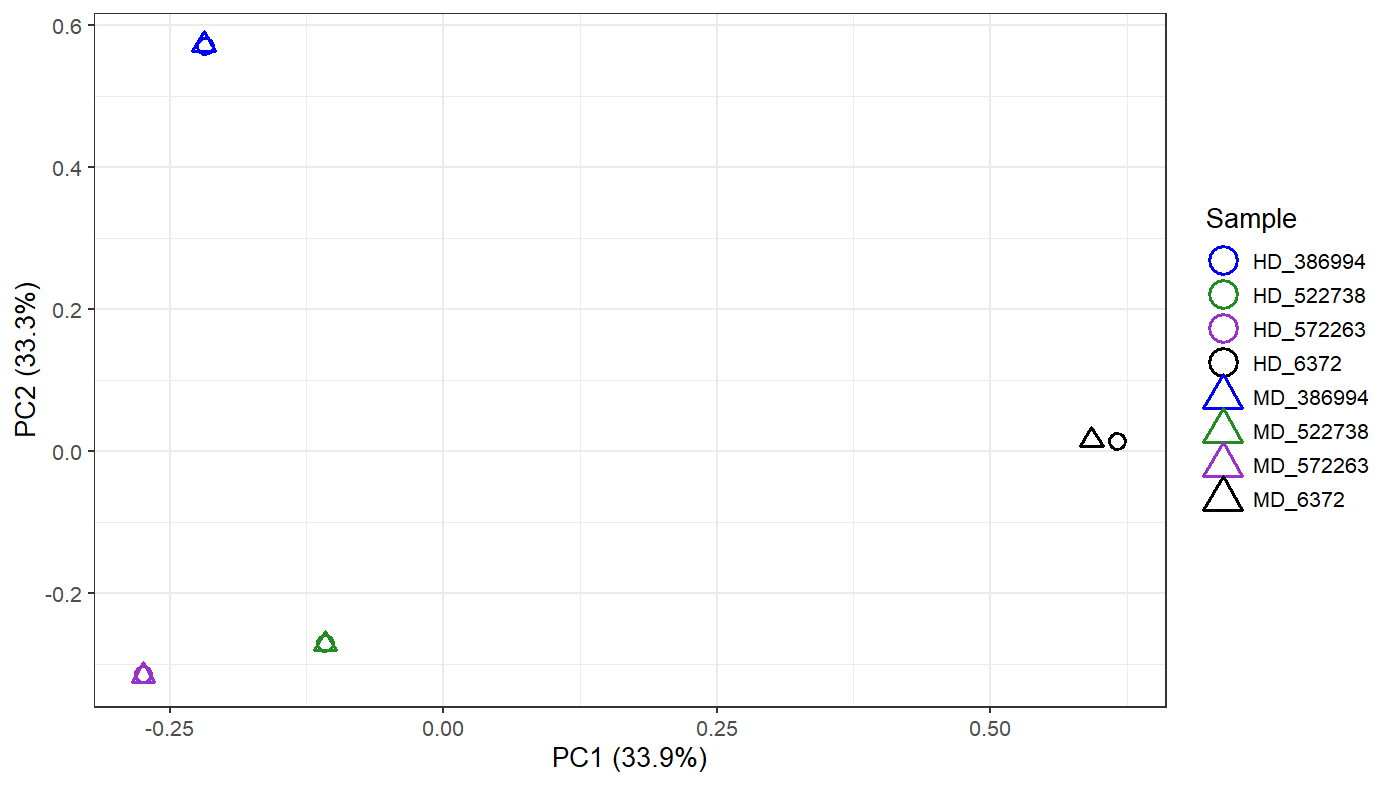


**Supplementary Figure S4. Probability of Identity for the OVSNP600 (a) and the OVSNP60 (b).** Probability of identity analysis for 469 individual deer sampled from 17 states in the US and Mexico and genotyped at 517,261 SNPs on the OVSNP600 array (a) and for 1,335 deer sampled from 18 states genotyped at 64,839 SNPs on the OVSNP60 array (b).

1. **OVSNP600** (b) **OVSNP60**


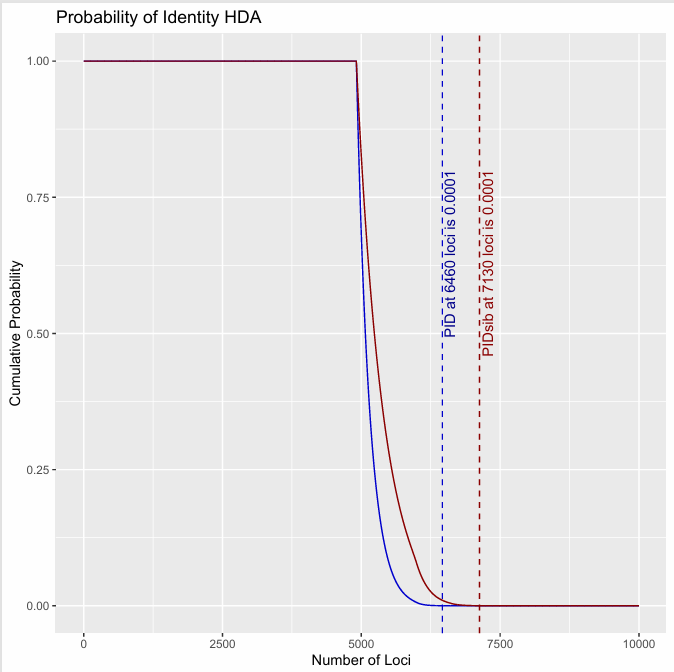

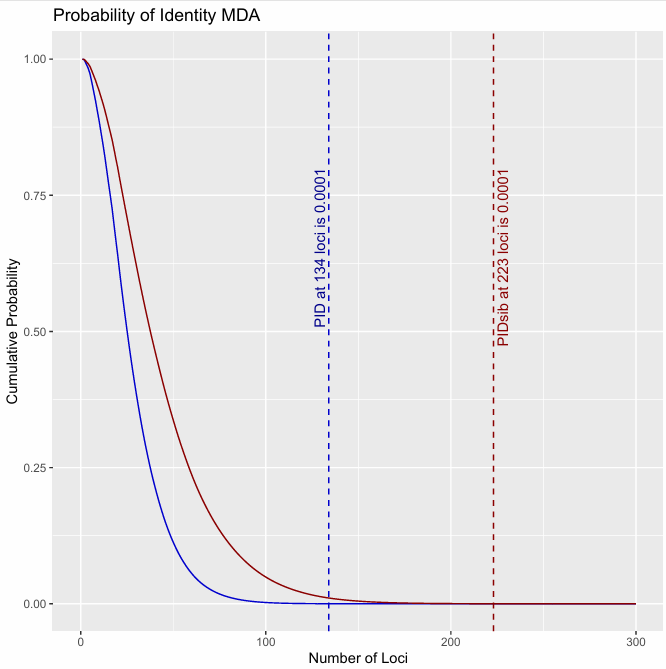


**Supplementary Figure S5. PCA of Michigan deer samples included on the OVSNP60 array.** Principal components analysis showing PC1 and PC2 for 145 individual Michigan deer genotyped at 64,132 SNPs on the OVSNP60 array. Samples are color-coded by location of origin (lower or upper peninsula of Michigan).


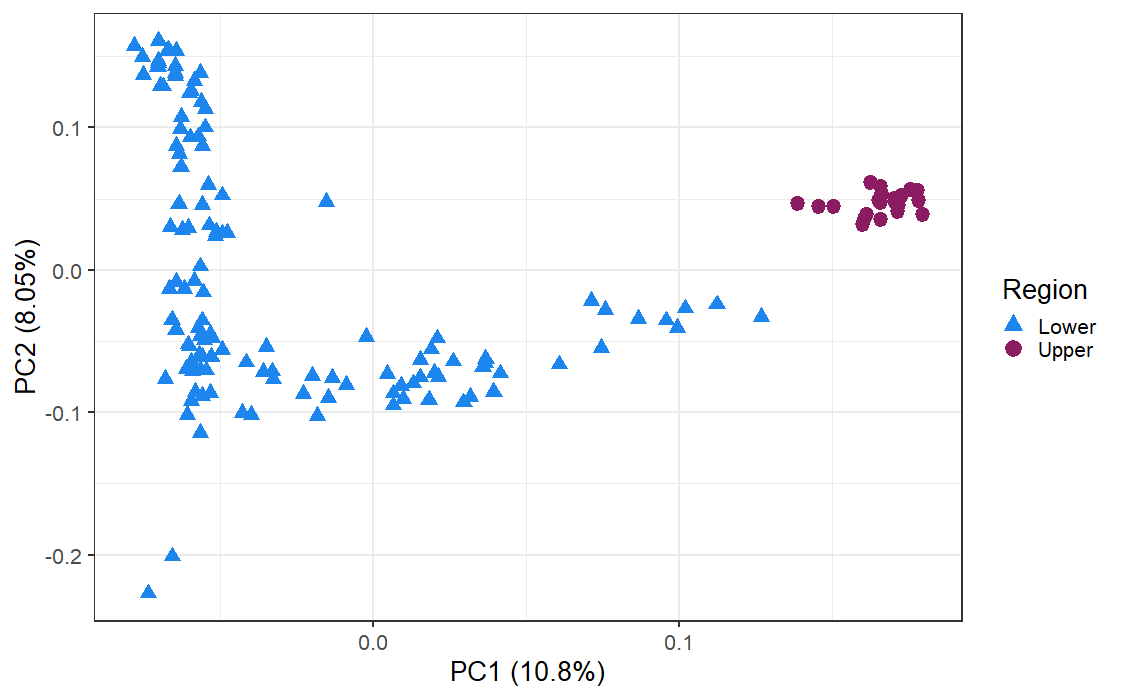


**Supplementary Figure S6. Cross-validation errors for OVSNP600 (a) and OVSNP60 (b).** Cross-validation errors for K=1 – 11 genetic clusters based on the OVSNP600 dataset (a) and for K=1 – 20 genetic clusters based on the OVSNP60 dataset (b). The optimal number of clusters was chosen as the one with the lowest cross-validation (K=5 for OVSNP600 and K=11 for OVSNP60).

(a) (b)


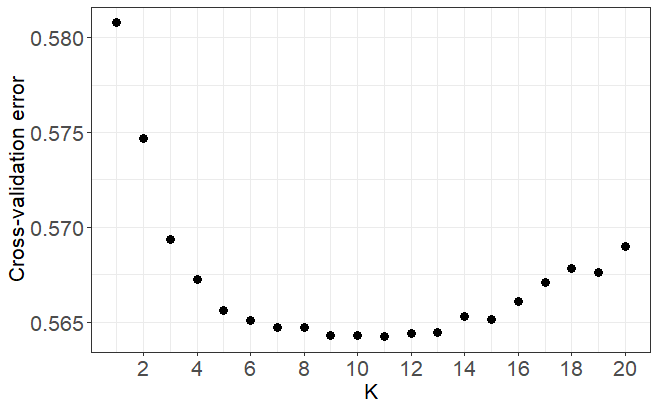

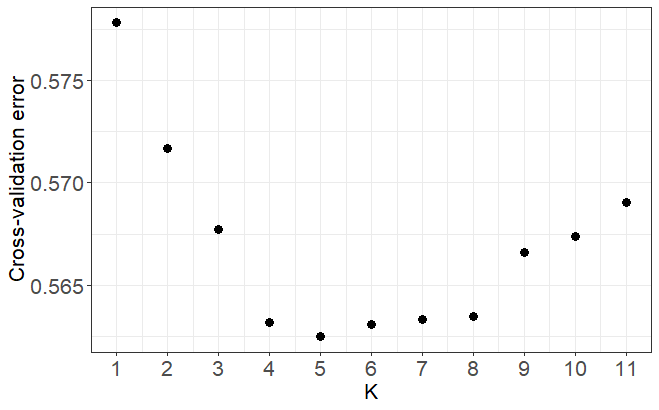


**Supplementary Figure S7.** **Admixture results for OVSNP600 at K=5.** Admixture analysis showing the optimal number of clusters (K=5) for 469 individual deer sampled from 17 states in the US and Mexico and genotyped at 517,261 SNPs on the OVSNP600 array.


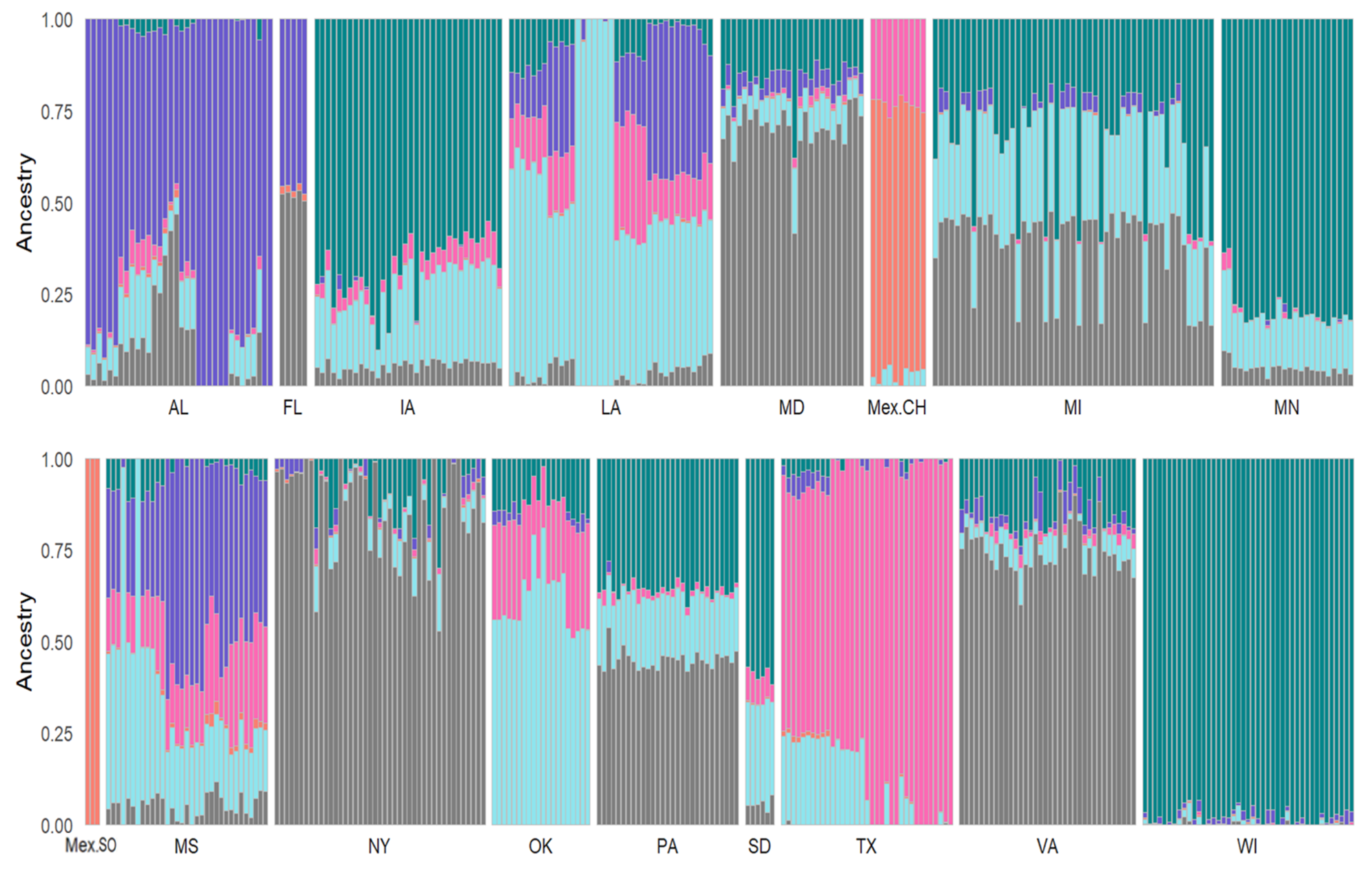

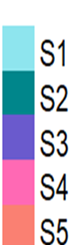


**Supplementary Figure S8. Admixture results for OVSNP60 at K=11.** Admixture analysis showing the optimal number of clusters (K=11) for 1,335 individual deer sampled from 18 US states and genotyped at 64,839 SNPs on the OVSNP60 array.
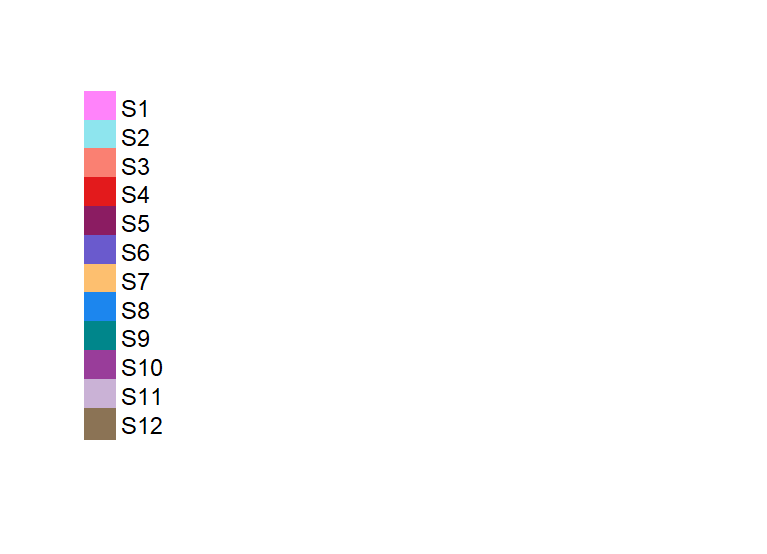

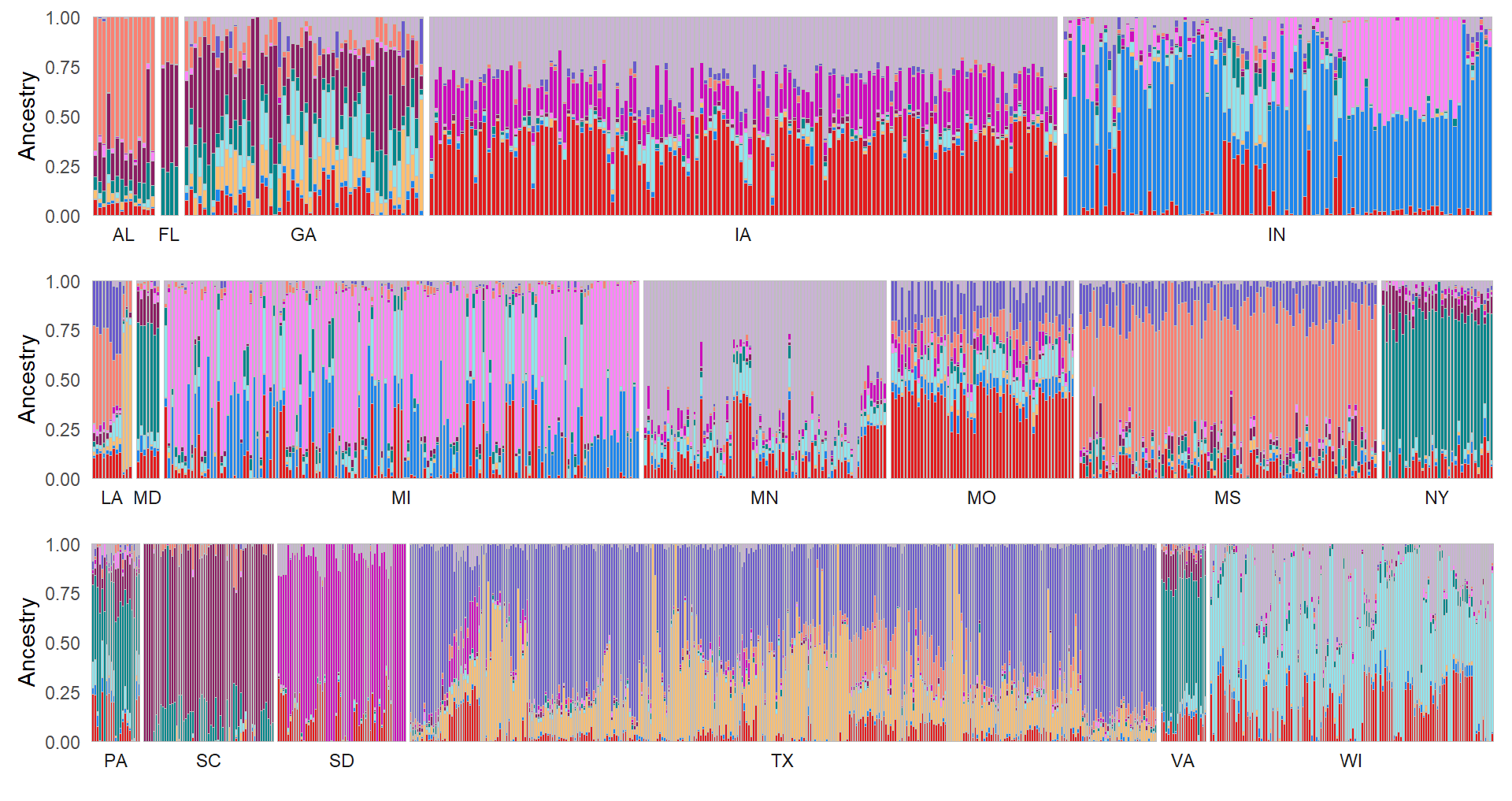

Supplement: Supplementary file 1 — Appendix S1: men70085‐sup‐0001‐AppendixS1.docx. [file MEN-26-e70085-s001.docx]
